# Assessing the Pathogenicity of *Berkeleyomyces rouxiae* and *Fusarium oxysporum* f. sp. *vasinfectum* on Cotton (*Gossypium hirsutum*) Using a Rapid and Robust Seedling Screening Method

**DOI:** 10.3390/jof10100715

**Published:** 2024-10-15

**Authors:** Andrew Chen, Duy P. Le, Linda J. Smith, Dinesh Kafle, Elizabeth A. B. Aitken, Donald M. Gardiner

**Affiliations:** 1 School of Agriculture and Food Sustainability, The University of Queensland, St. Lucia, QLD 4072, Australia; 2 New South Wales Department of Primary Industries, Narrabri, NSW 2390, Australia; duy.le@dpi.nsw.gov.au; 3 Ecosciences Precinct, Department of Agriculture and Fisheries, Dutton Park, QLD 4102, Australia; linda.smith@daf.qld.gov.au (L.J.S.); dinesh.kafle@daf.qld.gov.au (D.K.); 4 Queensland Alliance for Agriculture and Food Innovation, The University of Queensland, St. Lucia, QLD 4072, Australia

**Keywords:** upland cotton, pathogenicity, fungal effectors, Fusarium wilt, black root rot, *Secreted in Xylem* effector genes, endophyte, multilocus phylogeny

## Abstract

Cotton (*Gossypium* spp.) is the most important fibre crop worldwide. Black root rot and Fusarium wilt are two major diseases of cotton caused by soil-borne *Berkeleyomyces rouxiae* and *Fusarium oxysporum* f. sp. *vasinfectum* (*Fov*), respectively. Phenotyping plant symptoms caused by soil-borne pathogens has always been a challenge. To increase the uniformity of infection, we adapted a seedling screening method that directly uses liquid cultures to inoculate the plant roots and the soil. Four isolates, each of *B. rouxiae* and *Fov*, were collected from cotton fields in Australia and were characterised for virulence on cotton under controlled plant growth conditions. While the identities of all four *B. rouxiae* isolates were confirmed by multilocus sequencing, only two of them were found to be pathogenic on cotton, suggesting variability in the ability of isolates of this species to cause disease. The four *Fov* isolates were phylogenetically clustered together with the other Australian *Fov* isolates and displayed both external and internal symptoms characteristic of Fusarium wilt on cotton plants. Furthermore, the isolates appeared to induce varied levels of plant disease severity indicating differences in their virulence on cotton. To contrast the virulence of the *Fov* isolates, four putatively non-pathogenic *Fusarium oxysporum* (*Fo*) isolates collected from cotton seedlings exhibiting atypical wilt symptoms were assessed for their ability to colonise cotton host. Despite the absence of *Secreted in Xylem* genes (*SIX6*, *SIX11*, *SIX13* and *SIX14*) characteristic of *Fov*, all four *Fo* isolates retained the ability to colonise cotton and induce wilt symptoms. This suggests that slightly virulent strains of *Fo* may contribute to the overall occurrence of Fusarium wilt in cotton fields. Findings from this study will allow better distinction to be made between plant pathogens and endophytes and allow fungal effectors underpinning pathogenicity to be explored.

## 1. Introduction

Cotton (*Gossypium hirsutum*) is the most important fibre crop in the world. Approximately 25 million tons of cotton are produced worldwide per annum with an estimated value of USD 12 billion [1]. In Australia, the cotton industry is primarily based in the inland regions of New South Wales and southern Queensland, generating an annual revenue of AUD 1.9 billion [2].

Two important diseases of cotton in Australia are black root rot (BRR) and Fusarium wilt caused by *Berkeleyomyces rouxiae* and *Fusarium oxysporum* f. sp. *vasinfectum* (*Fov*), respectively [2,3]. Both fungal pathogens are considered hemibiotrophic plant pathogens. *B. rouxiae* was formerly known as *Thielaviopsis basicola* within the family Ceratocystidaceae [4]. *B. rouxiae* first colonises living root tissues and then consumes the root cells causing black discolouration, which results in brittle root tissues and reduced water and nutrient uptake by the plants [5]. Severe black root rot infection typically results in stunted seedlings, which can delay the flowering of the plants [6], but it does not often cause plant death in the cotton fields [3]. Yield losses of up to 10% caused by BRR have been reported in Australia [3].

*Fov* is a soil-borne pathogen that colonises the vascular system of the plants. Its proliferation inside the water-conducting vessels causes a blockage of the water supply to the upper parts of the plant, leading to leaf wilting, chlorosis, necrosis, vascular discolouration, plant stunting, defoliation, and plant death [7]. Globally, there are multiple races of *Fov*, but isolates originating from Australia belong to their own distinct lineage [8,9]. Vegetative compatibility groupings (VCGs) are also used to classify *Fov* isolates. Initially, in a study by Fernandez et al. (1994), ten VCGs were identified in *Fov* pathogenic to cotton, these being 0111-01110, with the first three digits representing *Fov* and the last number (1–10) identifying the VCG subgroup. They found that each of the races 1, 2, 3, 4, and 6 corresponded to a distinct VCG [10]. The Australian *Fov* isolates were then identified with distinct VCGs, 01111 and 01112 [11]. Studies showed that most isolates within the same VCG had the same rDNA and mtDNA haplotypes, although multiple haplotypes were observed in some cases. Overall, three ancestral lineages of *F. oxysporum* pathogenic towards cotton were observed, including race 3, which is common in Egypt, Sudan, and Israel and considered a single clone; the Australian biotypes; and all others including Californian races and race 1 from the Americas, races 7 and 8 from China, and race 4 from India [7].

In recent years, many isolates of *F. oxysporum* from cotton seedlings exhibiting atypical wilt symptoms have been identified, and they do not phylogenetically conform to the standard Australian biotypes [12]. This highlights a need to investigate further the potential of *F. oxysporum* to cause disease in cotton plants. There are currently a number of undetermined VCGs within *Fov* [13], as well as the presence of Australian isolates that are not compatible with VCG01111 and VCG0112 but never-the-less showed the ability to infect cotton plants [2,12]. This study aims to address whether minor or mildly virulent races of *Fov* may be contributing to the overall pathogenicity of the *F. oxysporum* populations in the cotton-growing regions of Australia.

Genomic resources are important for understanding the evolution of plant pathogens. At the same time, knowledge about specific effector genes can be translated to sensitive and accurate diagnostic tools to aid the detection of fungal diseases. This in turn contributes to an effective integrated disease management program. Genomic resources for *Berkeleyomyces* spp. are not well developed in the public domain. The only long read assembly that is currently available is a nanopore-based draft assembly (GenBank accession: GCA_026151695.1) created from a *B. rouxiae* strain that infects cannabis plants [14]. In this study, we aim to assess the virulence of *B. rouxiae* strains on cotton and, by doing so, identify a suitable strain from which a reference genome can be created.

Assessing the pathogenicity of these important cotton pathogens is critical in understanding not just virulence but also detecting new biotypes that may contribute to the evolution of pathogen populations across the wide cotton-growing regions.

## 2. Materials and Methods

### 2.1. Fungal Isolates

A total of 12 fungal isolates were used in this study. Four confirmed *Fusarium oxysporum* f. sp. *vasinfectum* isolates, namely, *Fov* SG1, *Fov* SG26, *Fov* SG55, and *Fov* TH1, were recovered from diseased stems with vascular browning, a typical characteristic of Fusarium wilt, and were collected during a cotton disease survey in regional Queensland (Table 1). *Fusarium oxysporum* isolates *Fo* BRF1, *Fo* BRF2, *Fo* SHF6, and *Fo* WRF2 were collected from hypocotyls of cotton seedlings in regional New South Wales (NSW). These seedlings displayed collar rot in the form of red-brown lesions around the seedling collars [12] (Table 1). The *Berkeleyomyces rouxiae* isolates were collected from the roots (StrB22, BRR4, and RVB4.1) and the crown vascular tissue (22BRR77) of cotton plants showing black root rot symptoms in regional NSW (Table 1). Using the single spore isolation method [3], monoconidial cultures for all 12 isolates were obtained from spores grown on cultures of half-strength potato dextrose agar (PDA) (*F. oxysporum*) or 10% carrot extract agar defined as carrot juice extracted from 100 g of carrots per litre of water (*B. berkeleyomyces*).

The diameters of the colonies were recorded and then used to normalise conidia production per unit area of the colony. Slides for microscopic examination were prepared using a tape touch method [15]. Fungal structures were examined under a Leica DM1000 compound microscope with an ICC50 W Camera using the 40× objective (Leica Camera, Wetzlar, Germany). *F. oxysporum* isolates were stained with lactophenol cotton blue before visualisation under the microscope.

To prepare a spore suspension, culture plates were flooded with 10 mL of sterile water. Mycelia and conidia were gently scraped off the plates using a sterile L-shaped disposable spreader (Thermo Fisher Scientific, Waltham, MA, USA). The suspension was collected, and the conidia concentration was determined using a hemacytometer (Merck, Darmstadt, Germany) and an appropriate dilution factor.

### 2.2. DNA Extraction, PCR, and Phylogenetic Analysis

DNA extraction was performed using mycelial scraped off a plate and then subjected to the microwave oven method [12]. PCR was performed using a 1 in 10 diluted DNA template and Dreamtaq (Thermo Fisher Scientific, Waltham, MA, USA). PCR amplicons of the correct size were confirmed on a 1% agarose gel, purified using a GeneJET PCR purification kit (Thermo Fisher Scientific, Waltham, MA, USA), and Sanger-sequenced (Australian Genome Research Facility, Melbourne, Australia).

Universal primers for *Secreted in Xylem* (*SIX*) genes 1 to 14 that specifically detect the presence or the absence of these effector genes in *Fusarium oxysporum* species complex were obtained from a previous study [16] (Table S1). *Fov*-specific primers for detecting *SIX6* in the Australian *Fov* biotypes were obtained from a previous study [17] (Table S1). PCR was performed to retrieve gene fragments of the translation elongation factor 1-alpha (TEF-1α), mitochondrial small subunit rDNA (mtSSU rDNA), nitrate reductase (*NIR*), and phosphate permease (*PHO*) using previously specified primers and conditions [9,18,19,20]. Minichromosome Maintenance Complex Component 7 (*MCM7*), the RNA polymerase II gene encoding the second-largest protein subunit (*RPB2*), was PCR amplified using primers derived from previous studies [21,22]. The primer sequences, along with the PCR conditions and amplicon sizes are listed in Table S1. The collection of sequences forming the backbone of the phylogenetic analysis was retrieved from the respective studies for *F. oxysporum* [2,9,18] and *B. rouxiae* [4]. Genbank accessions for these sequences, as well as for the ones generated in this study, are listed in Tables S2–S4.

Geneious Prime v 2024.0.7 (Biomatter Pty. Ltd., Auckland, New Zealand) was used to perform the phylogenetic analysis. DNA sequences were aligned using MAFFT v 7.490 [23] and edited over two iterations to remove gaps and derive a consensus alignment sequence. Bayesian inference was used to construct the phylogenetic trees using MrBayes v 3.2.6 [24]. The settings included the GTR-G-I model of substitution and two independent analyses on four Markov chain Monte Carlo (MCMC) chains for 2,000,000 generations, with a burn-in rate of 25% for every 1000 generations sampled. Orthologous gene sequences from other *Fusarium* spp. were used to anchor the whole phylogeny of the same gene. The tree branches were transformed into a cladogram and visualised in Geneious Prime.

### 2.3. Growth Chamber Experiments

Seeds of the cotton cv. ‘Sicot 746B3F’ were directly sown into 105 mm (width) × 105 mm (length) × 120 mm (depth) square pots. ‘Sicot 746B3F’ is resistant to Fusarium wilt, having a resistance F-rank of 135 (https://csd.net.au/disease-ranks/; accessed on 30 September 2024). The potting mix (UQ23) contained 70% composted pine bark of 0–5 mm in size and 30% Coco Peat and has a pH range of 5.5–6.5. UQ23 potting mix was then pasteurised using steam. For potting, each pot had three seeds that produced one to three seedlings per pot. Pots were lightly fertilised with a teaspoon of a balanced fertiliser (Osmocote). For each treatment, six pots containing seeds were put in a rectangular plastic tray with dimensions of 35 cm (length) × 29 cm (width) × 5.5 cm (depth). The trays had a layer of soil approximately 2–3 cm deep underneath the pots.

A double-tier Conviron growth chamber of model GEN1000 SH (Conviron, Winnipeg, MB, Canada) was used for plant growth. Plants for both *Fov* and *B. rouxiae* virulence testing were grown concurrently in the same chamber. A single tier can hold 5 trays of plants, and each tray represents a single treatment. The top tier contained the four *Fov* treatments, and the bottom tier contained the four *B. rouxiae* treatments (Figures S1 and S2). Each tier also had its own uninoculated controls (sterile water). The growth chamber conditions were set to 16 h of photoperiod with a day/night temperature of 26 °C/22 °C, respectively. Relative humidity was set to default at 60%. Plants were lightly watered every two to three days. The *F. oxysporum* experiment was set up later independent of the first experiment but in the same chamber with the same running conditions.

### 2.4. Plant Inoculation

For establishing the liquid cultures, four to five mycelial plugs were used to inoculate sterile liquid media. Half-strength potato dextrose broth (PBD, 500 mL) was used to culture each of the eight *F. oxysporum* isolates, whereas 10% carrot juice (200 mL) was used to culture the *B. rouxiae* isolates. Cultures were continuously shaken on a rotating platform at a speed of 120 rpm and a temperature of 26 °C for 7 days. The spore concentration of each culture was determined using a hemacytometer on the day of inoculation (Table S5). The *Fov* and *Fo* cultures produced spores in the range of 2.7 × 10^5^ to 3.8 × 10^7^ conidia per mL, whereas the *B. rouxiae* culture produced spores in the range of 1.8 × 10^5^ to 1.4 × 10^6^ condia per mL for all but StrB22, which produced a low number of spores but abundant mycelia (Table S5).

Unfiltered culture (500 mL) with the above-mentioned concentrations was used for root dipping. Firstly, 2-week-old cotton seedlings were uprooted from the pots (Figures S1 and S2). Their roots were cleaned by rinsing with distilled water and then blotted dry on paper towels. Plants were then root-dipped in the unfiltered culture for 5 min (Figures S1D and S2D). Soil from the pots of each treatment was tipped into a disposable bag and mixed evenly with 250 mL of the unfiltered culture before seedlings were re-potted. After inoculation, each tray was then bagged with a disposable (700 mm × 1000 mm) waste bag (Richlands, Australia) to avoid cross-contamination (Figures S1B and S2B).

### 2.5. Symptom Assessment and Pathogen Reisolation from Plant Tissues

Plants inoculated with *B. rouxiae* were examined at 15 days post inoculation (dpi). Plants inoculated with *Fov* were examined at 27 dpi. Due to the observation that some of these *Fov* inoculated plants were hitting the ceiling of the growth chamber at the time of harvest, in the subsequent growth chamber trial with *F. oxysporum*, plants were examined at an earlier stage, at 17 dpi.

Plants were examined for external discolouration on the roots and stems. Plant weight was determined using both the above and below-ground portions. Plant height was measured from the basal node to the terminal bud of the main stem. Root discolouration was measured as the length of discoloured roots and was expressed as a percentage of the total length of the roots. External stem discolouration was scored at a 5–10 cm region above the basal node of the main stem. Internal discolouration was assessed by longitudinally cutting the stems. Leaf wilting was scored as the number of leaves that were either dropped, wilting, or showing yellowing symptoms over the number of leaf nodes along the main stem, with or without leaves attached.

Koch’s postulate was performed to assess the extent of the colonisation by the pathogen in the host using a previously described method [25]. The plant tissues were surface sterilised in 70% ethanol for 60 s and were blotted dry under aseptic conditions. Roots (main root node and fine roots), stem (basal node of the stem and 7–10 cm above ground), and petiole (leaf nodes joining stem) tissues of approximately 5 mm × 2 mm pieces were embedded into half-strength PDA (*Fov* and *F. oxysporum*) or 10% carrot agar (*B. rouxiae*) containing 100 ppm streptomycin sulfate. Four segments per tissue type per plant were plated. Plates were incubated in the dark at 26 °C for 4 days and then scored for the presence of *F. oxysporum* and *B. rouxiae* colonies under a microscope. A positive was determined firstly by the presence of microconidia and mycelia (*F. oxysporum*) or endoconidia chains and aleuriospores (*B. rouxiae*). A visual identification was also made by comparing the morphology and colour of the colonies with those of the pure culture plates.

### 2.6. Statistical Analysis

Statistical analysis was performed in SPSS statistics for Macintosh v29 (IBM Corp, Armonk, NY, USA). A Shapiro–Wilk normality test was first performed to assess whether the dataset fits a normal distribution. A one-way analysis of variance (ANOVA) was then performed to derive the descriptive statistics, including the means and the 95% confidence interval of each treatment group. A homogeneity of variance was also assessed using Levene’s statistics. A post-hoc Tukey’s Honestly Significant Difference test was performed using sample-size harmonic means for unequal sample sizes to separate means for groups in homogeneous subsets.

## 3. Results

### 3.1. Morphology of the Isolates

**Figure 1:**
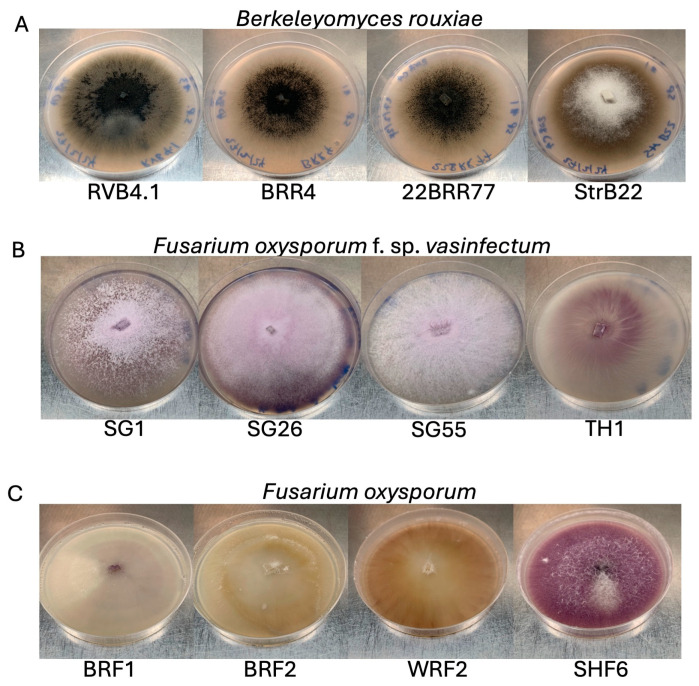
Colony morphology of *Berkeleyomyces rouxiae* and *Fusarium oxysporum* isolates used in this study. (**A**) *B. rouxiae* isolates RVB4.1, BRR4, 22BRR77, and StrB22 grown for 2 weeks on 10% carrot agar. (**B**) *Fusarium oxysporum* f. sp. *vasinfectum* isolates *Fov* SG1, *Fov* SG26, *Fov* SG55, and *Fov* TH1 grown for 2 weeks on half-strength potato dextrose agar (PDA). (**C**) *Fusarium oxsporum* isolates *Fo* BRF1, *Fo* BRF2, *Fo* WRF2, and *Fo* SHF6 grown for 2 weeks on half-strength PDA.

**Figure 2:**
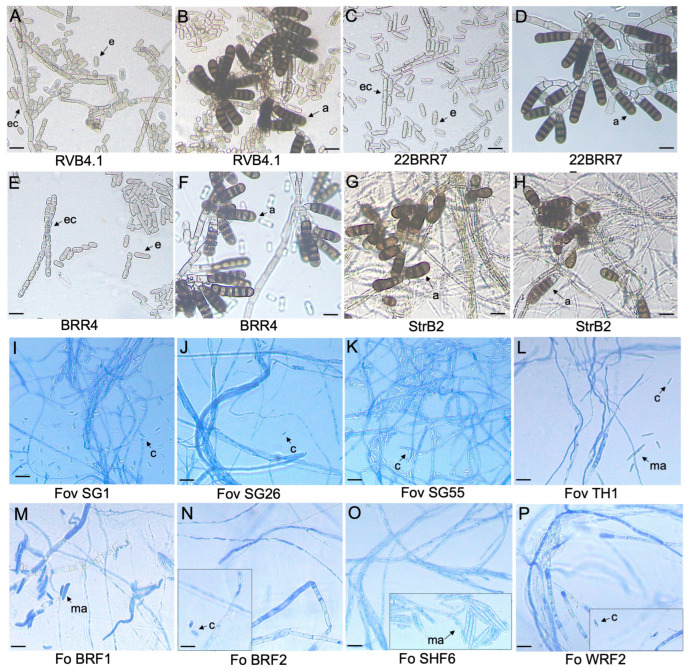
Compound microscopic images of *Berkeleyomyces rouxiae* and *Fusarium oxysporum* isolates used in this study. (**A**,**B**) *B. rouxiae* isolate RVB4.1. (**C**,**D**) *B. rouxiae* isolate 22BRR77. (**E**,**F**) *B. rouxiae* isolate BRR4. (**G**,**H**) *B. rouxiae* isolate StrB22. (**I**) *Fusarium oxysporum* f. sp. *Vasinfectum* isolate (*Fov*) SG1. (**J**) *Fov* isolate SG26. (**K**) *Fov* isolate SG55. (**L**) *Fov* isolate TH1. (**M**) *Fusarium oxsporum* (*Fo*) isolate BRF1. Inset: a cluster of conidia. (**N**) *Fo* isolate BRF2. Inset: microconidia. (**O**) *Fo* isolate SHF6. Inset: a cluster of macroconidia. (**P**) *Fo* isolate WRF2. Inset: microconidia. e = endoconidia; ec = endoconidia chains; a = aleuriospores; c = microconidia; ma = macroconidia. Bars indicate a scale of 15 µm.

On carrot agar, the *B. rouxiae* isolates were olive green initially and darkened over time (Figure 1A). The isolates had uniform colony margins. RVB4.1, 22BRR77, and BRR4 were flat in elevation. StrB22 appeared white in the centre, with raised elevation from aerial hyphae, and circular. BRR4 and StrB22 produced few spores in liquid media containing 10–20% (*w*/*v*) carrot juice (Table S5). All four *B. rouxiae* isolates grew well on half-strength PDA. On both half-strength PDA and 10% carrot agar, all four isolates showed the presence of endoconidia and aleuriospores under a microscope (Figure 2A–H). Endoconidia were unicellular, mostly hyaline, sometimes brown, cylindrical, and produced singularly or in chains. No endoconidia chains were observed in StrB22. Endoconidial size varied from 10.2 to 16.0 μm in length and 4.7 to 5.4 μm in width (Figure S3A). Aleuriospores were dark brown and formed laterally or terminally on hyphal branches in chains of 2–6 cylindrical segments (Figure 2A–H). The size of a single aleuriospore varied from 32.7 to 40.6 μm in length and 11.4 to 12.3 μm in width (Figure S3B). Conidia production on half-strength PDA ranged from 16.7 to 26,800 endoconidia per mm^2^ colony (Figure S3C).

The *Fov* colonies appeared pale to light pink on half-strength PDA plates (Figure 1B), whereas the *F. oxysporum* colonies appeared to vary in colour, which included white (*Fo* BRF1), pale yellow (*Fo* BRF2), orange (*Fo* WRF2), and dark pink (*Fo* SHF6) (Figure 1C). For *Fov* SG1, *Fov* SG26, *Fov* SG55, and *Fo* SH6, white aerial hyphae were observed, whereas the mycelia of *Fov* TH1, *Fo* BRF1, *Fo* BRF2, and *Fo* WRF2 were flat and closely adhered to the media. Microconidia were on average 6.0–12.4 μm in length and 2.4–2.7 μm in width (Figure S4A). They were hyaline, single-celled, oval to reniform, and were abundantly produced in false heads on short monophialides (Figure 2I–P). Macroconidia were on average 20–30 μm long, generally three septate, hyaline, sometimes slightly curved, and had an apical hook (Figure 2I–P). Conidia production on half-strength PDA ranged from 300 to 65,460 endoconidia per unit area of the colony (Figure S4B).

### 3.2. Phylogenetic Analysis Confirms the Australian Isolates as Belonging to Berkeleyomyces rouxiae

**Figure 3:**
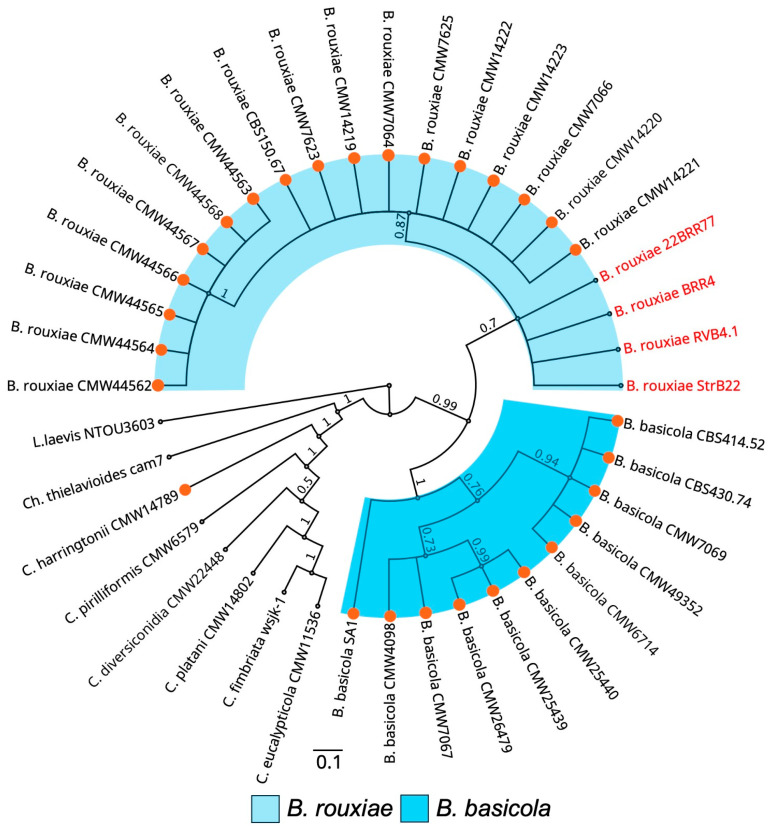
Phylogenetic positions of *Berkeleyomyces rouxiae* isolates within the genus *Berkeleyomyces* determined using Bayesian inference. Phylogenetic relationships were reconstructed using concatenated sequences of *MCM7* and *RPB2* genes. Isolates examined in this study are highlighted in red. Orange circles indicate accessions that were used to define the genus *Berkeleyomyces* [4]. The bar indicates a scale range of 0.1. Node values show the posterior probability. Genus is abbreviated as Ch. for *Chalaropsis*, C. for *Ceratocystis*, B. for *Berkeleyomyces*, and L. for *Lignincola*.

Bayesian inference analysis based on the concatenated sequences of *MCM7* and *RPB2* genes showed that all four *B. rouxiae* isolates clustered within the *B. rouxiae* subclade of the genus *Berkeleyomyces* (Figure 3). The subclade of *B. rouxiae* was phylogenetically separated from that of the closely related species, *B. basicola*. The phylogenetic positions of our strains confirmed their *B. rouxiae* identity.

### 3.3. Phylogenetic Analysis and SIX Gene Profiles Place the New Fov Isolates with Other Australian Fov but Suggest the Fo Isolates Are Unique

**Figure 4:**
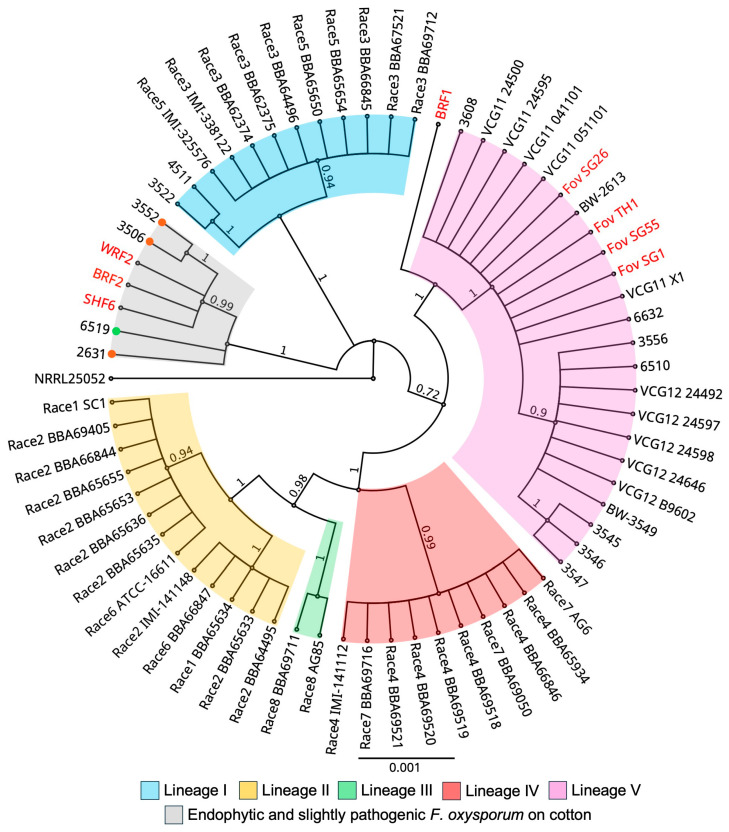
Bayesian phylogeny of the *Fusarium oxysporum* isolates inferred from combined analysis of translation elongation factor, mitochondrial small subunit rDNA, nitrate reductase, and phosphate permease gene sequences. *Fusarium oxysporum* f. sp. *vasinfectum* isolates are classified into lineages I–V [7,9]. Isolates examined in this study are highlighted in red. The endophytic and slightly pathogenic *F. oxysporum* isolates from cotton were classified as a distinct lineage [18], and these isolates were characterised to be either non-pathogenic (green circle) or slightly pathogenic (orange circles) on cotton plants. VCG01111 and VCG01112 are abbreviated as VCG11 and VCG12, respectively. The bar indicates a scale range of 0.001. Node values show the posterior probability.

To resolve the phylogenetic positions of the *F. oxysporum* isolates within the pathogen forms that infect cotton, Bayesian analysis was performed using concatenated partial sequences of TEF-1α, mtSSU rDNA, *PHO*, and *NIR* gene sequences. The *Fov* representative isolates belonging to distinct lineages I to V were retrieved from previous studies [9,18] and were used to anchor this phylogeny (Figure 4). The topology of the phylogeny can be correlated with race and geographic origin. Lineage I contains mostly race 3 strains from Egypt and race 5 from Sudan. Lineage II contains race 1 and 2, primarily from the United States, along with race 6 from Brazil. Lineage III contains race 8 strains from China. Lineage IV has strains of race 4 and race 7 from India and China, respectively. Lineage IV is represented by the Australian biotypes, namely, VCG01111 and VCG01112 (Figure 4). An additional lineage, independent of lineage I to V, was identified in another study [18], and it contained *F. oxysporum* isolates that were endophytic or slightly pathogenic on cotton plants.

With the lineages of *Fov* correctly defined by the topology of phylogenetic tree, the four *Fov* isolates, namely, *Fov* SG1, *Fov* SG26, *Fov* SG55, and *Fov* TH1, were phylogenetically positioned within lineage V (Figure 4). They grouped amongst the VCG01111 isolates while further distinctions allowed VCG01112 isolates to form a subclade within lineage V. Of the 4 *Fusarium oxysporum* isolates, *Fo* BRF1 descended from an ancestral split from lineage V, suggesting that it shares the same evolutionary descent as lineage V isolates (Figure 4). The divergence event separated the lineage that gave rise to *Fo* BRF1 from the lineage that gave rise to lineage V. On the other hand, *Fo* BRF2, *Fo* SHF6, and *Fo* WRF2 were phylogenetically clustered within a lineage that is independent of lineages I to V (Figure 4). Within this phylogroup, there are other *F. oxysporum* strains isolated from the rhizosphere of wild relatives of cotton in Australia, and they appeared to cause little to no Fusarium wilt symptoms on cotton plants [18].

**Figure 5:**
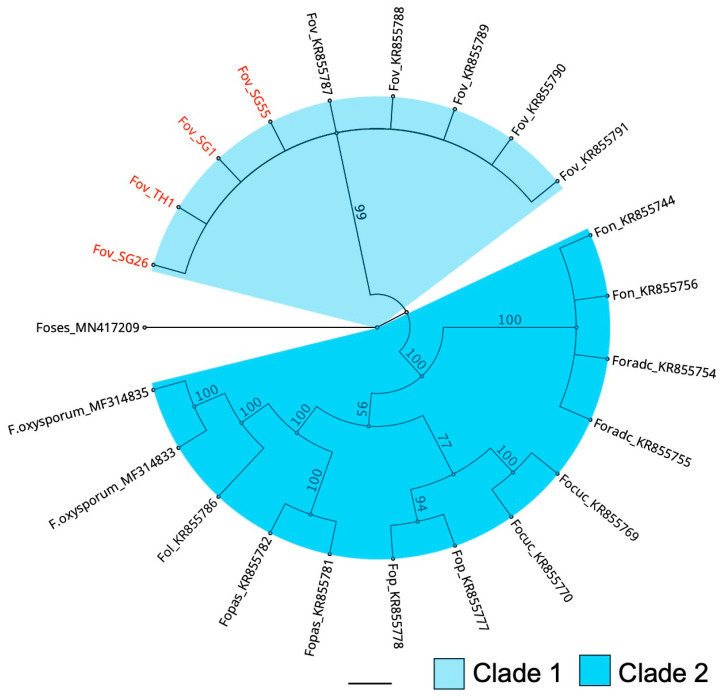
Bayesian inference phylogenetic reconstruction of *Secreted in Xylem 6* gene (*SIX6*) in *Fusarium oxysporum* f. sp. *vasinfectum* isolates and isolates from other *formae speciales* of *Fusarium oxysporum*. Isolates examined in this study are highlighted in red. *Focuc* = *Fusarium oxysporum* f. sp. *cucumerinum*; *Fol* = *Fusarium oxysporum* f. sp. *lycopersici*; *Fopas* = *Fusarium oxysporum* f. sp. *passiflora*; *Fon* = *Fusarium oxysporum* f. sp. *niveum*; *Fop* = *Fusarium oxysporum* f. sp. *pisi*; *Foradc* = *Fusarium oxysporum* f. sp. *radicis-cucumerinum*; *Foses* = *Fusarium oxysporum* f. sp. *sesami*. Bars indicate a scale range of 0.05. Node values show the posterior probability.

To ascertain whether any of the *F. oxysporum* isolates were *Fov*, we used the marker *FovSIX6* that specifically detects the presence of *SIX6* in the Australian *Fov* biotypes [17]. *FovSIX6* was detected in *Fov* SG1, *Fov* SG26, *Fov* SG55, and *Fov* TH1, but the primers did not amplify a product in *Fo* BRF1, *Fo* BRF2, *Fo* SHF6, and *Fo* WRF2 (Table 2). To further validate these results, the *FovSIX6* gene products were sequenced and then used to construct a Bayesian inference phylogeny comprised of *SIX6* gene sequences of other pathogen forms of *F. oxysporum* (Figure 5). The sequences used as the backbone of this phylogeny were retrieved from a previous study [26]. *Fov*-specific *SIX6* genes showed that the *Fov* isolates presented here and other *Fov* representatives formed a distinct lineage that is separated from the rest of the *F. oxysporum* strains (Figure 5). This confirmed that our *Fov* isolates included in this study belongs to the Australian biotypes.

To confirm the absence of *SIX6* in the *F. oxysporum* isolates and to generate an effector gene profile for each *SIX* gene, we performed a diagnostic PCR screen on these isolates using a set of universal primers for *SIX1*–*SIX14*. These primers were developed in a previous study [16]. The results showed that *Fov* SG1, *Fov* SG26, *Fov* SG55, and *Fov* TH1 possessed *SIX11*, *SIX13,* and *SIX14* in addition to *SIX6* (Table 2). The effector profiles of these *Fov* isolates are consistent in comparison to those of the other *Fov* accessions (BRIP) characterised in the previous study [16]. None of the *SIX* genes were detected in *Fo* BRF1, *Fo* BRF2, *Fo* SHF6, and *Fo* WRF2, suggesting that they are most likely absent in these isolates (Table 2).

### 3.4. Berkeleyomyces rouxiae Isolates Showed Variability in Disease Severity on Cotton

**Figure 6:**
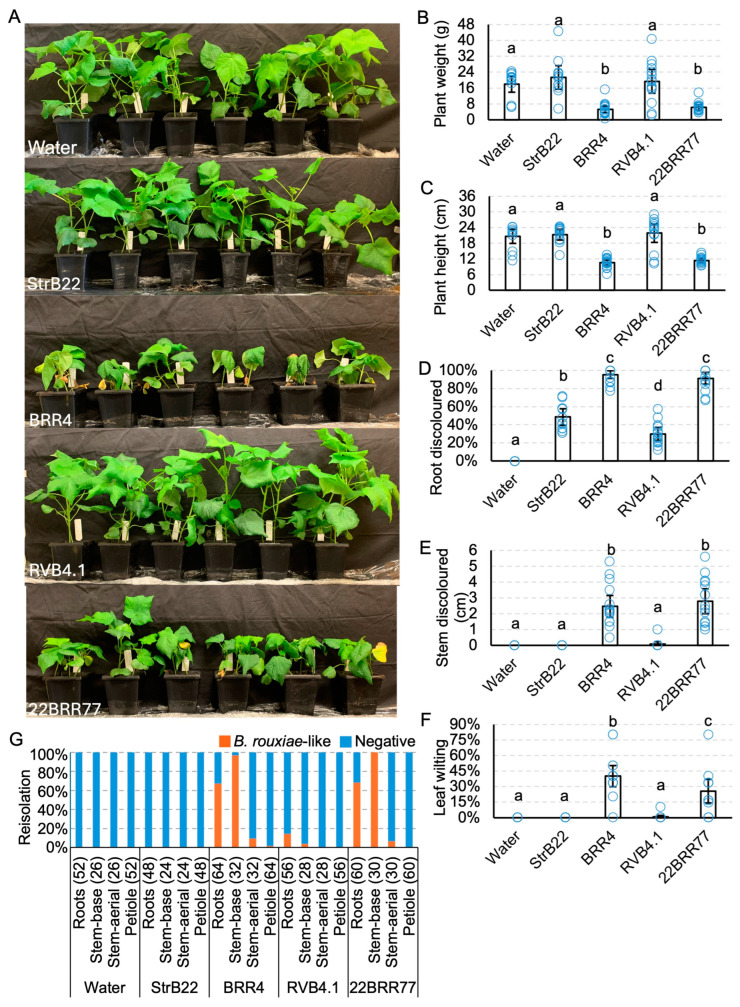
Virulence testing of *Berkeleyomyces rouxiae* isolates on cotton cv. Sicot746 B3F. (**A**) Plants at harvest (15–20 days post inoculation). (**B**) Total (above and below ground) plant weight. (**C**) Plant height. (**D**) Percentage of roots discoloured. (**E**) Total stem discoloured. (**F**) Percentage of leaves wilted or dropped. (**G**) Reisolations of *B. rouxiae*-like colonies on half-strength potato dextrose agar from the roots, stems, and petioles of uninoculated plants and plants inoculated with the *B. rouxiae* isolates. Numbers indicate the number of samples assayed per tissue type. Letters indicate a significant separation of means by Tukey’s range test at *p* = 0.05. Error bars indicate a 95% confidence interval.

**Figure 7:**
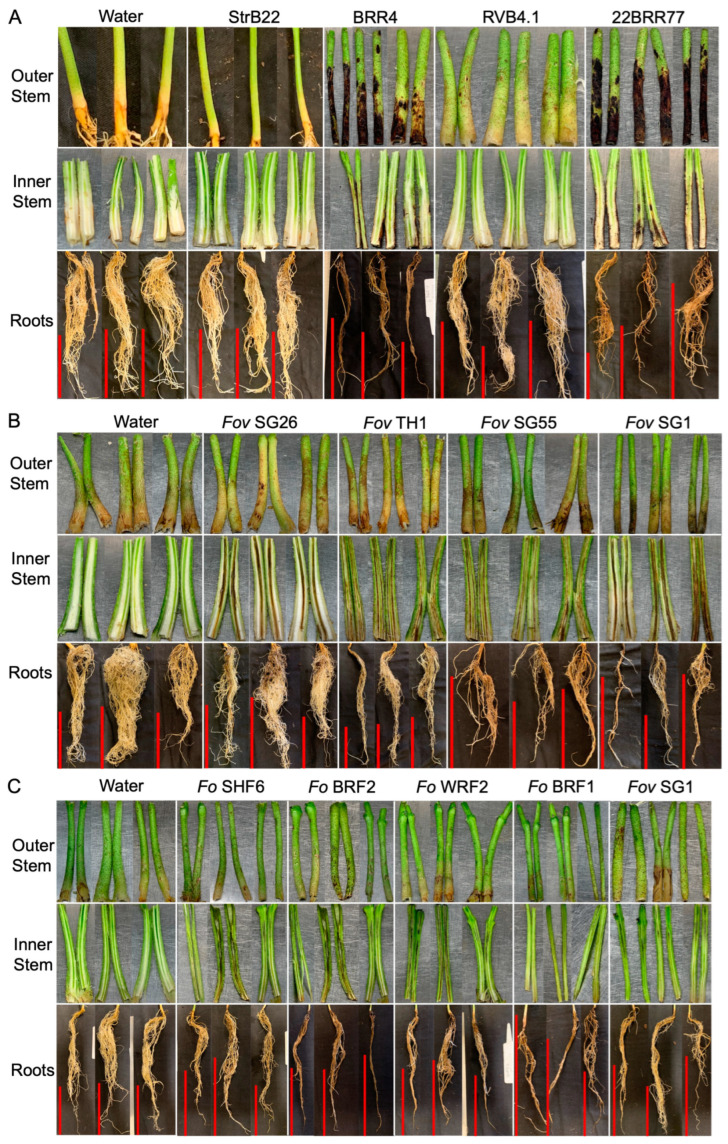
Symptomatology of *Berkeleyomyces rouxiae* and *Fusarium oxysporum* isolates on cotton cv. Sicot746 B3F at harvest. (**A**) Stem and root symptomatology of Sicot746 B3F plants inoculated with *B. rouxiae* isolates StrB22, BRR4, RVB4.1 and 22BRR77. (**B**) Stem and root symptomatology of Sicot746 B3F plants inoculated with *Fusarium oxysporum* f. sp. *vasinfectum* (*Fov*) isolates *Fov* SG26, *Fov* TH1, *Fov* SG55 and *Fov* SG1. (**C**) Stem and root symptomatology of Sicot746 B3F plants inoculated with *F. oxysporum* (*Fo*) isolates *Fo* SHF6, *Fo* BRF2, *Fo* WRF2, *Fo* BRF1 and *Fov* SG1 (positive control). Plants inoculated with water served as a negative control. Red vertical bars indicate a scale of 10 cm.

Sicot746 B3F plants inoculated with *B. rouxiae* were assessed for both internal and external symptoms at 15 dpi (Figure 6). The isolates 22BRR77 and BRR4 significantly reduced the plant height and weight of Sicot746 B3F when compared with the uninoculated controls (Figure 6A–C and Figure 7A). These plants also developed black lesions on the epidermis of stems at 1–6 cm above the basal node (Figure 7A). Internal discolouration was observed in the vascular tissues of some of the stems. Furthermore, the roots of 22BRR77 and BRR4 inoculated plants appeared blackened and stunted, a characteristic symptom of black root rot (Figure 7A). The leaves of these plants also displayed significant wilt symptoms (Figure 6A,F). Reductions in weight and height were negatively associated with black discolouration in the lower stems of the plants and the roots of the symptomatic plants (Figure 6D,E and Figure 7A). These symptoms appeared to develop uniformly amongst infected plants (Figures S5–S7). Plants inoculated with StrB22 and RVB4.1 did not have any visible external symptoms (Figure 6, Figures S5 and S6), and their plant height and weight were comparable to that of the uninoculated controls (Figure 6B,C). The roots of these plants were still discoloured but to a lesser extent when compared to 22BRR77 and BRR4-inoculated plants Figure 6D, Figure 7A, Figures S5 and S6).

The reisolation of *B. rouxiae* from *B. rouxiae*-inoculated plants on 10% carrot agar plants produced characteristic dark colonies, although not all of them were identified as positives (Figure S8). Root reisolation frequencies of 67% and 68.3% were observed for plants inoculated with 22BRR77 and BRR4, respectively (Figure 6G). The reisolation frequency was higher in the stem than in the roots, with 96.9% and 100% of the basal stem issues being identified as positives for 22BRR77 and BRR4--inoculated plants, respectively (Figure 6G). Above-ground stems and petiole tissues showed less than 10% reisolation frequency for 22BRR77 and BRR4. The reisolation frequency for RVB4.1 was less than 10% for all tissue types assayed. No positives were identified from StrB22-inoculated plants or uninoculated control plants. The conidial concentration of StrB22 was 10^3^-fold lower than that of the other *B. rouxiae* isolates (Table S5). These observations collectively suggest that the inoculation using StrB22 was not successful.

### 3.5. Isolates of Fusarium oxysporum f. sp. vasinfectum Varied in Their Virulence towards Cotton

**Figure 8:**
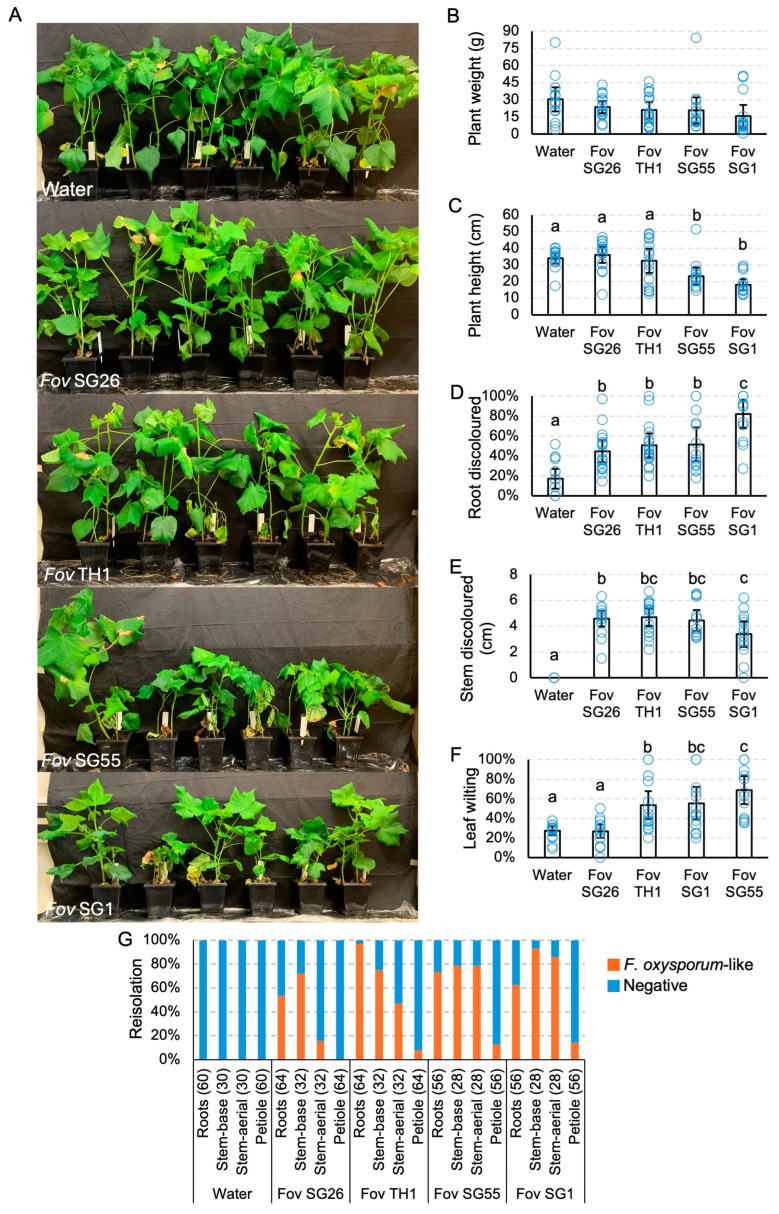
Virulence testing of *Fusarium oxysporum* f. sp. *vasinfectum* (*Fov*) isolates on cotton cv. Sicot746 B3F. (**A**) Plants at harvest (27–34 days post inoculation). (**B**) Total (above and below ground) plant weight. (**C**) Plant height. (**D**) Percentage of roots discoloured. (**E**) Total stem discoloured. (**F**) Percentage of leaves wilted or dropped. (**G**) Reisolations of *F. oxysporum*-like colonies on half-strength potato dextrose agar from the roots, stems, and petioles of uninoculated plants and plants inoculated with the *Fov* isolates. Numbers indicate the number of samples assayed per tissue type. Letters indicate a significant separation of means by Tukey’s range test at *p* = 0.05. Error bars indicate a 95% confidence interval.

*Fov*-inoculated plants did not show any significant differences in plant weight at harvest (Figure 8B). However, *Fov* SG1 and *Fov* SG55-inoculated plants were significantly shorter than uninoculated plants and *Fov* TH1 and *Fov* SG26-inoculated plants (Figure 8C). A brown discolouration was observed at the basal stem and in the roots of plants inoculated with the *Fov* isolates (Figure 7B and Figure 8D,E), but these symptoms were only sporadically detected (Figures S9–S11). However, these effects were significant when compared to the uninoculated controls (Figure 8D,E). *Fov* SG1-inoculated plants had 82% of roots discoloured, which was significantly higher than the plants inoculated with the other isolates (Figure 8D). *Fov* SG1, *Fov* SG55, and *Fov* TH1-inoculated plants also showed significantly higher levels of leaf wilting when compared to that of the uninoculated controls or plants inoculated with *Fov* SG26 (Figure 8F). Internally, vascular discolouration, which is a characteristic symptom of Fusarium wilt, was consistently identified in the main root nodes and the cut stems of all *Fov*-inoculated plants (Figures S9–S11). Disease incidence, expressed as the number of plants carrying internal stem discolouration over the total number of plants assessed, was estimated at 100% for *Fov* SG1 and *Fov* SG26, 92.8% for *Fov* SG55, and 43.7% for *Fov* TH1.

Tissue reisolations produced white to light pink colonies that were further confirmed to be *F. oxysporum*-like under a microscope (Figure S8). Reisolation frequencies were high in both the roots (53.1–96.9%) and lower stems (71.9–92.9%) for all *Fov* inoculated plants (Figure 8G). The upper stem reisolation frequency was the highest in *Fov* SG1-inoculated plants (85.7%) and lowest in *Fov* SG26-inoculated plants (15.6%). The presence of *Fov* at the base of the petiole was detected at a low reisolation frequency (7.8–14.3%) in plants inoculated with all except *Fov* SG26 (Figure 8G).

### 3.6. Virulence of Fusarium oxysporum on Cotton

**Figure 9:**
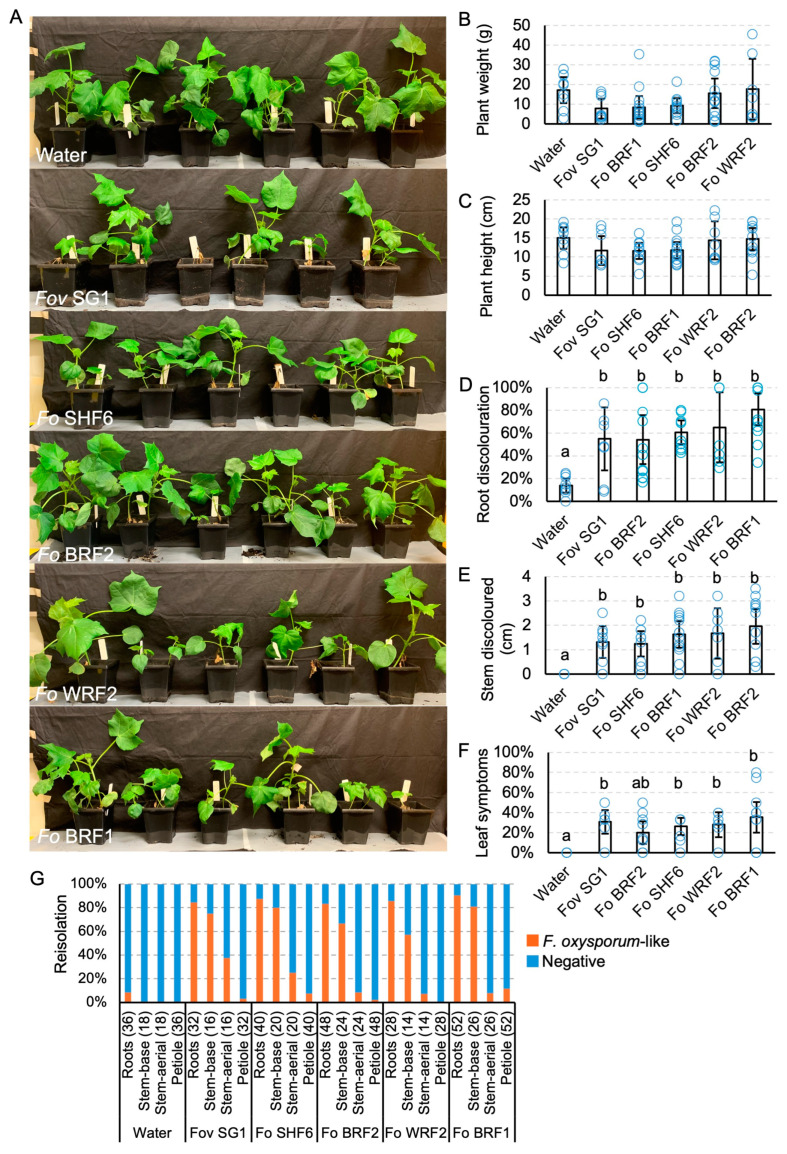
Virulence testing of *Fusarium oxysporum* (*Fo*) isolates on cotton cv. Sicot746 B3F. (**A**) Plants at harvest (17–22 days post inoculation). (**B**) Total (above and below ground) plant weight. (**C**) Plant height. (**D**) Percentage of roots discoloured. (**E**) Total stem discoloured. (**F**) Percentage of leaves wilted or dropped. (**G**) Reisolations of *F. oxysporum*-like colonies on half-strength potato dextrose agar from the roots, stems, and petioles of uninoculated plants and plants inoculated with the *Fov* isolates. Numbers indicate the number of samples assayed per tissue type. Letters indicate a significant separation of means by Tukey’s range test at *p* = 0.05. Error bars indicate a 95% confidence interval.

Plants inoculated with the four *F. oxysporum* isolates were harvested at 17 dpi, which means that the plants were a lot younger than the *Fov*-inoculated plants at harvest (27 dpi) (Figure 9A). No significant differences in plant height and weight were detected between plants inoculated with the *F. oxysporum* isolates and the uninoculated controls (Figure 9B,C). Light brown discolouration in the roots and the stems was evident (Figure 9D,E). Internally, black discolouration in the vascular tissues of the stems was also observed (Figure 9C). These effects are significant when compared to uninoculated controls (Figure 9D,E), although the symptoms were not consistently identified amongst the plants of each treatment group (Figures S12–S14). Based on the internal discolouration of the stem, *F. oxysporum*-inoculated plants showed disease incidences at 71.4% for *Fo* WRF2, 60% for *Fo* SHF6, 58.3% for *Fo* BRF2, and 30.8% for *Fo* BRF1. The inoculated plants also showed significantly elevated leaf wilting when compared to the uninoculated controls (Figure 9F).

Reisolation from plants inoculated with the *F. oxysporum* isolates showed that the colour of recovered colonies was comparable to that of the pure culture (Figure S8). Reisolation frequencies were 83.3–90.4% from the roots, 57.1–80.8% from the lower stem regions, and 7.1–25% from the upper stem regions (Figure 9G). *F. oxysporum*-like colonies were only recovered at a low frequency (2.5–11.5%) from the petioles of *Fo* BRF1, *Fo* SHF6, and *Fo* BRF2-inoculated plants.

## 4. Discussion

The pathogenicity of black root rot pathogen relied on using naturally or artificially infested soil or commercial potting mix, respectively [27]. On the other hand, there is an array of methods such as root dip [28], soil drench [29], stem puncture, and agar plug amended potting mix [30] to assess the pathogenicity of cotton-*Fusarium* spp. Different inoculation methods also resulted in different implications. Therefore, in this study, we sought to assess the virulence of both black root rot pathogen and cotton-*Fusarium* spp. using a rapid and robust seedling and soil inoculation assay and a spore suspension obtained from unfiltered liquid cultures. This inoculation method was successful in differentiating the virulence of both *B. rouxiae* and *Fusarium* spp. on cotton. Using unfiltered suspension for root dipping and soil inculcation, the virulence of *B. rouxiae* was visually detected at 15 dpi in comparison to 19 to 30 dpi using artificially or naturally infested soil [27]. Similarly, screening for Fusarium wilt typically takes 1–3 months for disease phenotypes to be expressed and assessed in different plant species [31,32,33]. In our study, we were able to terminate and assess the aggressiveness of cotton-*Fusarium* spp. as soon as 15 dpi. Given that ‘Sicot 746B3F’ is a Fusarium wilt resistant cultivar, the level of symptoms observed on these plants was consistent with a high disease severity. Thus, the number of spores used to inoculate a plant host can be adjusted to reflect the sensitivity of the host to a specific fungal pathogen. A seedling screening system such as this can be scaled up to provide preliminary assays for pathogen virulence at a high throughput. We acknowledge that we have only tested a limited number of isolates in this study. Expanding the number of test isolates or optimising the conditions to test other plant pathosystems could further improve the robustness of this screening system. A reliable screening system is important in determining the host range of specific plant pathogens and the genetics of plant and pathogen interactions.

Spores and mycelia were not filtered before being applied to the plants and the soil. Given that spore production varies significantly amongst the different *Fov* and *Berkeleyomyces* isolates (Figures S3 and S4), a pre-determined number of spores per pot can still lead to variations in the build-up of inoculum for infection. Instead, sufficient spores and mycelia were applied to the soil at a high density. Fine roots and the new root growth in soil saturated with the fungus can achieve good infection rates, thus providing uniformity in infection levels. Furthermore, plant growth under a controlled environment with a set of constant parameters can reduce variability in growth and varied disease expression due to environmental effects. However, a varied number of spores and mycelia in the inoculum applied may also contribute to varied disease severity.

The result shows that *B. rouxiae* BRR4 and 22BRR77 were clearly pathogenic towards cotton. The discolourations in the stems were distinct and appeared more severe when compared to the discolouration levels in the roots (Figure 7). This contrasts the pot trial of another study in which root discolouration appeared to be the most dominant trait associated with the black root rot of cotton [3]. In that study, *Berkeleyomyces* spore culture was mixed with the potting mix, and seeds are directly sown into the *Berkeleyomyces*-infested soil. This inoculation method mimics the field conditions in which residual *Berkeleyomyces* spores in the soil provide a natural inoculum for the germinating cotton seeds.

In plants severely affected by 22BRR77, some discolouration was observed in the vascular regions of the stems. However, black root rot infection is commonly observed on the epidermis of below-ground hypocotyls and roots. Vascular infection associated with *Berkeleyomyces* is rarely detected in Australia. However, it has been shown that in the presence of root-knot nematodes, *B. basicola* can induce necrosis within the vascular tissues of cotton plants [34]. Therefore, open lesions and wounds caused by nematodes or wounds such as those artificially induced during root dipping and washing in our study can potentially lead to the movement of *B. rouxiae* through the vasculature in cotton plants. Nevertheless, both BRR2 and 22BRR77 were only recovered at a low level in the arial parts of the plants, suggesting that their ability to infect plants is primarily through the roots and lower parts of the plants. The strong discolouration on the stem surface could be used as a trait marker to identify and confirm Berkeleyomyces infections in the cotton field. Despite being identified as *B. rouxiae*, RVB4.1 did not have any effects on plant growth, suggesting that there is variability in the ability of *B. rouxiae* strains to infect specific plant species.

Variation in the virulence of *Berkeleyomyces rouxiae* on cotton has not been observed so far in field surveys at five locations in NSW [3]. In this study, RVB4.1 and its asymptomatic appearances on cotton, when compared to the virulent strains BRR4 and 22BRR77, is what we believe to be the first ever report of strain-specific variation in the virulence of black root rot on cotton hosts. Therefore, the research moving forward will focus on using comparative genomics to characterise genomic regions that may explain the underlying virulence on cotton. Small effector proteins that are secreted by the fungus during infection on cotton will also be predicted and compared.

DNA sequences have been used to investigate the evolutionary relationships among the *Fov* races. Based on multiple conserved gene sequences, different races of *Fov* were separated into five distinct lineages [7,9,16,35]. In our study, the four *Fov* isolates conformed to the other Australian *Fov* biotypes in lineage V, specifically within a group of VCG01111 isolates, while further distinctions allowed VCG01112 isolates to form a subclade within this lineage. In a recent field study, of the 389 isolates collected from 10 geographical locations across the major cotton growing regions in Australia, 94% or 366 isolates conferred to the Australian biotypes [2]. Furthermore, of the 166 *Fov* isolates subjected to the VCG analysis, 147 isolates were assigned to VCG01111, while only one isolate was confirmed as VCG01112. These results collectively suggest that VCG01111 is the dominant VCG in Australia and that it has adapted to a broad range of geographic locations. The genetic diversity associated with the international *Fov* isolates has not been detected in the Australian cotton fields. Since the disease incidences of Fusarium wilt have risen steadily over the years, it will be interesting to compare the virulence of the *Fov* isolates with that of ‘historical’ *Fov* isolates dating back 10–15 years

Interestingly, 23 *F. oxysporum* isolates from the field surveys were obtained from cotton plants showing Fusarium wilt symptoms but were not clustered with the Australian biotypes and did not possess the FovSIX6 marker [2]. Similarly, it was shown that non-Australian *Fov* isolates are a part of lineage V and can be distinguished phylogenetically from the Australian isolates [35]. Taken together, these results support the proposal that the different lineages of *Fov* are independently evolving [10], highlighting a need to further investigate the diversity of *Fov* isolates beyond the current classification system. Virulence testing using these isolates could also help us to understand their contributions to the pathogenicity of *Fov* populations in the field.

All four *F. oxysporum* isolates were isolated from the hypocotyls of cotton seedlings displaying the collar rot disease [12]. They were not initially identified as *Fov* due to the fact that the plants the isolates were obtained from did not display Fusarium wilt symptoms. Effector gene profiling based on the presence or absence of *SIX* genes has been used to differentiate the different pathogen forms of *F. oxysporum* as well as *F. oxysporum* endophytes in a previous study [16]. By using this diagnostic system, it was determined that none of the four *F. oxysporum* isolates used in this study possessed any *SIX* genes. Despite not having any *SIX* genes, these isolates were able to colonise the vascular tissues within the stems and cause disease on cotton plants. These results suggest that they behave in a manner characteristic of a vascular wilt pathogen. To identify and characterise genome regions unique to *Fov* and *Fo*, pac-bio sequencing is currently being undertaken for *Foz* SG1 and *Fo* BRF1.

*Fo* BRF1 shares the same evolutionary descent as lineage V but appeared to form a lineage that is independent of lineage V. Similarly, *Fo* BRF2, *Fo* SHF6, and *Fo* WRF2 were clustered within a lineage that is independent of the *Fov* lineages. This lineage was previously defined by *F. oxysporum* isolates that were endophytic or slightly pathogenic on cotton cultivars [18]. Despite having reduced virulence on cotton cultivars, the authors observed that these isolates were highly pathogenic on a wild relative of cotton, therefore suggesting that these cotton hosts could serve as an inoculum reservoir for the co-occurrence of these ‘wild’ *Fov* populations in the field [18]. Moreover, selection pressures exerted by the changing environment and *Fov* resistance in cotton cultivars may have favoured the selection of the ‘wild’ *Fov* populations with enhanced virulence. The four *F. oxysporum* isolates used in this study were part of a collection consisting of 186 *F. oxysporum* isolates recovered from cotton seedlings showing the collar rot disease [12]. Fusarium wilt symptoms were not observed on these plants. Out of this collection, only 21.5% of the isolates grouped with the Australian biotype lineage V while 78.5% of the isolates clustered with the other lineages [12]. Our results are consistent with these findings. Taken together, these results collectively suggest that under disease-inducive conditions, *F. oxysporum* strains can act opportunistically on cotton plants, and their ability to colonise and exert virulence on hosts is dependent on the host genotypes. The specificity of the Fusarium–cotton interactions may have been encoded by the cotton hosts. Thus, the *F. oxysporum* populations may have been enriched for mildly virulent strains that are capable of causing *Fov*-like symptoms on cotton plants in the field. They apparently lack the *SIX* gene effectors and are part of lineages that are independent of the *Fov* phylogroups.

## 5. Conclusions

Black root rot and Fusarium wilt are significant diseases constraining cotton production worldwide. Here, we adopted a plant screening system where the virulence of these pathogens can be assessed quickly and effectively on cotton plants. Our results contribute towards understanding the pathogenicity of these pathogen populations in the cotton fields and allow strategies in surveillance and detection to be improved.

## Figures and Tables

**Table 1:** Fungal isolates used in this study indicating their origins and date of collection.

Isolates ^1^	Species Name	Location	Cotton Tissue Sampled ^2^	Collection Date (d/m/y)
*Fov* SG1	*Fusarium oxysporum* f. sp. *vasinfectum*	Saint George, Queensland	Stem	20 April 2022
*Fov* SG26	*Fusarium oxysporum* f. sp. *vasinfectum*	Saint George, Queensland	Stem	20 April 2022
*Fov* SG55	*Fusarium oxysporum* f. sp. *vasinfectum*	Saint George, Queensland	Stem	21 April 2022
*Fov* TH1	*Fusarium oxysporum* f. sp. *vasinfectum*	Theodore, Queensland	Stem	31 December 2022
*Fo* BRF1	*Fusarium oxysporum*	Walgett, New South Wales	Hypocotyls	21 December 2017
*Fo* BRF2	*Fusarium oxysporum*	Walgett, New South Wales	Hypocotyls	21 December 2017
*Fo* SHF6	*Fusarium oxysporum*	Wee Waa, New South Wales	Hypocotyls	21 December 2017
*Fo* WRF2	*Fusarium oxysporum*	Mungindi, New South Wales	Hypocotyls	21 December 2017
RVB4.1	*Berkeleyomyces rouxiae*	Hillston, New South Wales	Roots	06 December 2017
StrB22	*Berkeleyomyces rouxiae*	Mungindi, New South Wales	Roots	06 December 2017
BRR4 (DAR85827)	*Berkeleyomyces rouxiae*	Condobolin, New South Wales	Roots	06 December 2017
22BRR77	*Berkeleyomyces rouxiae*	Wee Waa, New South Wales	Crown	02 June 2022

^1^ All isolates were archived and maintained locally, except for BRR4 isolate, which was deposited in the NSW Plant Pathology and Mycology Herbarium with the accession number DAR85827. ^2^ Diseased cotton plant parts where the pathogens were recovered.

**Table 2:** *SIX* gene profiles of the isolates used in this study.

Isolate	*SIX1*	*SIX2*	*SIX3*	*SIX4*	*SIX5*	*SIX6*	*SIX6* (*Fov*)	*SIX7*	*SIX8*	*SIX* *9-1*	*SIX* *10*	*SIX* *11*	*SIX* *12*	*SIX* *13*	*SIX* *14*
*Fov* SG1 ^1^	—	—	—	—	—	+	+	—	—	—	—	+	—	+	+
*Fov* SG26 ^1^	—	—	—	—	—	+	+	—	—	—	—	+	—	+	+
*Fov* SG55 ^1^	—	—	—	—	—	+	+	—	—	—	—	+	—	+	+
*Fov* TH1 ^1^	—	—	—	—	—	+	+	—	—	—	—	+	—	+	+
*Fo* BRF1 ^1^	—	—	—	—	—	—	—	—	—	—	—	—	—	—	—
*Fo* BRF2 ^1^	—	—	—	—	—	—	—	—	—	—	—	—	—	—	—
*Fo* SHF6 ^1^	—	—	—	—	—	—	—	—	—	—	—	—	—	—	—
*Fo* WRF2 ^1^	—	—	—	—	—	—	—	—	—	—	—	—	—	—	—
NRRL25433 ^2^	—	—	—	—	—	—	n/t	—	—	—	—	—	—	+	—
BRIP63607 ^2^	—	—	—	—	—	+	n/t	—	—	—	—	+	—	+	+
BRIP43351 ^2^	—	—	—	—	—	+	n/t	—	—	—	—	+	—	+	+
BRIP25374 ^2^	—	—	—	—	—	+	n/t	—	—	—	—	+	—	+	+
BRIP43344 ^2^	—	—	—	—	—	+	n/t	—	—	—	—	+	—	+	+
BRIP43336 ^2^	—	—	—	—	—	+	n/t	—	—	—	—	+	—	+	+
BRIP43339 ^2^	—	—	—	—	—	+	n/t	—	—	—	—	+	—	+	+
BRIP43356 ^2^	—	—	—	—	—	+	n/t	—	—	—	—	+	—	+	+

^1^ *SIX* gene products for these isolates were amplified using universal primers based on conserved coding regions of the *SIX* genes [16], except for *SIX6* (*Fov*), which used primers that can specifically detect *SIX6* in Australian *Fov* isolates [17]. ^2^ The *SIX* gene profiles of these *Fov* isolates were obtained from a previous study [16]. — indicates absence of the *SIX* gene amplicon. + indicates the presence of the *SIX* gene amplicon. n/t abbreviates ‘not tested’.

## Data Availability

The original contributions presented in the study are included in the article/Supplementary Materials; further inquiries can be directed to the corresponding authors. Gene sequences generated in this study have been deposited in Genbank and can be accessed under BioProject PRJNA1162925.

## Supplementary Materials

The following supporting information can be downloaded at https://www.mdpi.com/article/10.3390/jof10100715/s1. Figure S1: Plant growth before and after inoculation with *Berkeleyomyces rouxiae* isolates; Figure S2: Plant growth before and after inoculation with *Fusarium oxysporum* f. sp. *vasinfectum* isolates; Figure S3: The spore size and growth rates of *Berkeleyomyces rouxiae* isolates after 3 weeks of growth on half-strength PDA plates; Figure S4: The spore size and growth rates of *Fusarium oxysporum* isolates after seven days of growth on half-strength PDA plates; Figures S5–S7: Symptomatology of Sicot746 B3F seedlings inoculated with *Berkeleyomyces rouxiae* at harvest (15–20 days post inoculation); Figure S8: Reisolation of the *Berkeleyomyces rouxiae* and *Fusarium oxysporum* isolates from cotton plants on half-strength PDA plates (reverse view); Figures S9–S11: Symptomatology of Sicot746 B3F seedlings inoculated with *Fusarium oxysporum* f. sp. *vasinfectum* at harvest (27–34 days post inoculation); Figures S12–S14: Symptomatology of Sicot746 B3F seedlings inoculated with *Fusarium oxysporum* spp. at harvest (17–22 days post inoculation). Table S1: Primers used for PCR in this study; Table S2: *MCM7* and *RPB2* gene sequences used for phylogenetic analyses in this study; Table S3: TEF-1α, mtSSU rDNA, *PHO*, and *NIR* gene sequences used for phylogenetic analyses in this study; Table S4: *SIX6* gene sequences used for phylogenetic analysis in this study; Table S5: Spore concentrations of the liquid cultures determined on the day of inoculation. References [3,4,9,16,17,18,19,20,21,22,26] are cited in the Supplementary Materials.
