# Supplementary material for: Assessing the Pathogenicity of Berkeleyomyces rouxiae and Fusarium oxysporum f. sp. vasinfectum on Cotton (Gossypium hirsutum) Using a Rapid and Robust Seedling Screening Method
Source: J Fungi (Basel). 2024 Oct 15;10(10):715. doi: 10.3390/jof10100715 (PMC11508790; doi:10.3390/jof10100715)
Supplement: Supplementary file 1 [file jof-10-00715-s001.zip › jof-3232262-supplementary.pdf]

Supplemental material

Assessing the pathogenicity of *Berkeleyomyces rouxiae* and *Fusarium oxysporum* f. sp. *vasinfectum* on cotton (*Gossypium hirsutum*) using a rapid and robust seedling screening method

Andrew Chen [1,*], Duy P. Le [2], Linda J. Smith [3], Dinesh Kafle [3], Elizabeth A. B. Aitken [1,*] and Donald M. Gardiner [4,*].

Table S1. Primers used for PCR in this study.

Primer name	5' to 3' sequence	Product (bp)	PCR[1] (C)	Reference
Cer-MCM7F	ACICGIGTITCIGAYGTNAAGCC	628	55	de Beer et al., 2014
Cer-MCM7R	TTRGCAACACCAGGRTCACCCAT			
RPB2-5Fb	GAYGAYCGTGATCACTTYGG	1129	55	Fourie et al., 2015
RPB2-7Rb	CCCATRGCYTGYTTRCCCAT			
PHO 1	ATCTTCTGGCGTGTTATCATG	620	50	Skovgaard et al., 2001
PHO 4	GTGCTGGAAGAAGTCTCTCC			
MS 1	CAGCAGTCAAGAATATTAGTCAATG	400	52	White et al., 2000
MS 21	CTCTCCTCCTCAAGTACTGC			
EF-1	ATGGGTAAGGAAGACAAGAC	652		O'Donnell et al., 1998
EF-2	GGAAGTACCAGTGATCATGTT			Skovgaard et al., 2001
FovSIX6-F2	CTTCACGGCAGACCCG	~467	55	Chakrabarti et al., 2011
SIX6-R1	CAAGACCAGGTGTAGGCATT			
SIX1-F	TCTCCATTACTTTGTCTCACG	694-733	55	Czislowski et al., 2021
SIX1-R	CGATTTAGGCGATTCGGGG			
SIX2-F	GGTTCCCATCGTTGAAGC	327-330	55	Czislowski et al., 2021
SIX2-R	TTGGTTTAAATCTGCGTGTC			
SIX3-F	TTACTACGAGCTTCAGCACC	223	55	Czislowski et al., 2021
SIX3-R	GCATTAGGTGTTGCAACAGG			
SIX4-F	CAGCTCAGACAGTCAGCC	~491	55	Czislowski et al., 2021
SIX4-R	GGCCTTGAGTCGAATGAGC			
SIX5-F	TCA TCA GTA CTG TGC TTG CC	347-354	55	Czislowski et al., 2021
SIX5-R	CAT GTT GAG TCT GCT CCT CC			
SIX6-F	CTCTCGAGACACSCTTCC	396-399	52	Czislowski et al., 2021
SIX6-R	GATCCACCAATACCTTCAT			
SIX7-F	GAGGTGACATTTGACATCACC	113	55	Czislowski et al., 2021

SIX7-R	TAGTATGCGCGCCATTGG			
SIX8-F	CCCTAGCCGTCTCTGTGGC	163-165	60	Czislowski et al., 2021
SIX8-R	CGTTCGACAAGGGCTCTCTCG			
SIX9-G1-F	TTCAAGTCGGTTGCTACGC	118	55	Czislowski et al., 2021
SIX9-G1-R	GCATCCCAAAATCCAAAGCG			
SIX10-F	TCACGTTTCGAGTTGGTCC	202	55	Czislowski et al., 2021
SIX10-R	ACACCAAATCGAGTCGATGC			
SIX11-F	GTTGCTCCTCCTTTGCTGG	163	56	Czislowski et al., 2021
SIX11-R	TACCACTCTGACCAGTCACC			
SIX12-F	CAGAATGCTTGTGTGTGTGG	171	56	Czislowski et al., 2021
SIX12-R	ATCACCAGAGCATGAACCCC			
SIX13-F	TCTGATCAGCCTCCTAGCG	840	58	Czislowski et al., 2021
SIX13-R	CCACTGTAACTCGGCATCGA			
SIX14-F	TGTCTCAGCGTATCCTCGGC	147-197	58	Czislowski et al., 2021
SIX14-R	ATTCAGTGACAACGGGACCG			

[1] The annealing temperatures used in PCR.

Table S2. MCM7 and RPB2 gene sequences used for phylogenetic analyses in this study. They include these retrieved from the previous studies (Nel et al., 2018; Duy et al., 2022) as well as the ones generated in this study.

Species Name	Isolate name	MCM7	RPB2
Berkeleyomyces rouxiae	StrB22	PQ346555	PQ346543
Berkeleyomyces rouxiae	RVB4.1	PQ346556	PQ346544
Berkeleyomyces rouxiae	BRR4	PQ346557	PQ346545
Berkeleyomyces rouxiae	22BRR77	PQ346558	PQ346546
Berkeleyomyces rouxiae	CBS150.67	MF967107	MF967160
Berkeleyomyces rouxiae	CMW7623	MF967110	MF967193
Berkeleyomyces rouxiae	CMW14219	MF967086	MF967165
Berkeleyomyces rouxiae	CMW7064	MF967081	MF967187
Berkeleyomyces rouxiae	CMW7625	MF967112	MF967194
Berkeleyomyces rouxiae	CMW14222	MF967087	MF967168
Berkeleyomyces rouxiae	CMW14223	MF967111	MF967169
Berkeleyomyces rouxiae	CMW7066	MF967083	MF967189
Berkeleyomyces rouxiae	CMW14220	MF967103	MF967166
Berkeleyomyces rouxiae	CMW14221	MF967113	MF967167
Berkeleyomyces rouxiae	CMW44562	MF967089	MF967175
Berkeleyomyces rouxiae	CMW44564	MF967091	MF967177
Berkeleyomyces rouxiae	CMW44565	MF967092	MF967178
Berkeleyomyces rouxiae	CMW44566	MF967093	MF967179
Berkeleyomyces rouxiae	CMW44567	MF967094	MF967180
Berkeleyomyces rouxiae	CMW44568	MF967095	MF967181
Berkeleyomyces rouxiae	CMW44563	MF967090	MF967176
Berkeleyomyces basicola	CBS414.52	MF967104	MF967163
Berkeleyomyces basicola	CBS430.74	MF967101	MF967164
Berkeleyomyces basicola	CMW7069	MF967085	MF967191
Berkeleyomyces basicola	CMW49352	MF967102	MF967183

Berkeleyomyces basicola	CMW7067	MF967084	MF967190
Berkeleyomyces basicola	CMW25440	MF967088	MF967172
Berkeleyomyces basicola	CMW25439	MF967099	MF967171
Berkeleyomyces basicola	CMW6714	MF967079	MF967186
Berkeleyomyces basicola	SA1	MF967108	MF967198
Berkeleyomyces basicola	CMW26479	MF967105	MF967173
Berkeleyomyces basicola	CMW4098	MF967078	MF967174
Chalaropsis thielavioides	cam7	ON055141	ON055135
Ceratocystis pirilliformis	CMW6579	KM495453	KJ601630
Ceratocystis platani	CMW14802	KM495454	KJ601628
Ceratocystis eucalypticola	CMW11536	KM495428	KJ601612
Ceratocystis fimbriata	wsjk-1	MN990348	1MN957791
Ceratocystis diversiconidia	CMW22448	KJ601536	KJ601608
Ceratocystis harringtonii	CMW14789	KM495435	MF967170
Lignincola laevis	NTOU3603	KC417327	DQ836886

Table S3. TEF-1α, mtSSU rDNA, PHO, and NIR gene sequences used for phylogenetic analyses in this study. They include these retrieved from the previous studies (Skovgaard et al., 2001; Wang et al., 2010) as well as the ones generated in this study.

Species name	Isolate name	TEF-1α	mtSSU rDNA	PHO	NIR
Fusarium oxysporum f. sp. *vasinfectum*	SG1	PQ346535	PQ321298	PQ346559	PQ346547
Fusarium oxysporum f. sp. *vasinfectum*	SG55	PQ346538	PQ321301	PQ346562	PQ346550
Fusarium oxysporum f. sp. *vasinfectum*	TH1	PQ346539	PQ321302	PQ346563	PQ346551
Fusarium oxysporum f. sp. *vasinfectum*	SG26	PQ346542	PQ321305	PQ346566	PQ346554
Fusarium oxysporum	WRF2	PQ346536	PQ321299	PQ346560	PQ346548
Fusarium oxysporum	SHF6	PQ346537	PQ321300	PQ346561	PQ346549
Fusarium oxysporum	BRF2	PQ346540	PQ321303	PQ346564	PQ346552
Fusarium oxysporum	BRF1	PQ346541	PQ321304	PQ346565	PQ346553
Fusarium oxysporum	2631	EU246562	EU246597	EU246668	EU246634
Fusarium oxysporum	3506	EU246563	EU246598	EU246669	EU246635
Fusarium oxysporum	3552	EU246566	EU246600	EU246671	EU246637
Fusarium oxysporum	6519	EU246569	EU246603	EU246674	EU246640
Fusarium oxysporum f. sp. *vasinfectum*	3522	EU246564	EU246599	EU246670	EU246636
Fusarium oxysporum f. sp. *vasinfectum*	IMI-325576	EU246572	EU246606	EU246677	EU246643
Fusarium oxysporum f. sp. *vasinfectum*	IMI-338122	EU246573	EU246607	EU246678	EU246644
Fusarium oxysporum f. sp. *vasinfectum*	BBA62374	AF362142	AF362175	AF362236	AF362208
Fusarium oxysporum f. sp. *vasinfectum*	BBA62375	AF362143	AF362176	AF362237	AF362209
Fusarium oxysporum f. sp. *vasinfectum*	BBA64496	AF362159	AF362192	AF362253	AF362225
Fusarium oxysporum f. sp. *vasinfectum*	BBA65650	AF362154	AF362187	AF362248	AF362220
Fusarium oxysporum f. sp. *vasinfectum*	BBA65654	AF362155	AF362188	AF362249	AF362221
Fusarium oxysporum f. sp. *vasinfectum*	BBA66845	AF362153	AF362186	AF362247	AF362219
Fusarium oxysporum f. sp. *vasinfectum*	BBA67521	AF362152	AF362185	AF362246	AF362218
Fusarium oxysporum f. sp. *vasinfectum*	BBA69712	AF362162	AF362195	AF362256	AF362228
Fusarium oxysporum	4511	EU246567	EU246601	EU246672	EU246638
Fusarium oxysporum f. sp. *vasinfectum*	AG6	EU246547	EU246594	EU246665	EU246631
Fusarium oxysporum f. sp. *vasinfectum*	BBA65934	AF362165	AF362198	AF362259	AF362231
Fusarium oxysporum f. sp. *vasinfectum*	BBA66846	AF362164	AF362197	AF362258	AF362230

Fusarium oxysporum f. sp. *vasinfectum*	BBA69050	AF362156	AF362189	AF362250	AF362222
Fusarium oxysporum f. sp. *vasinfectum*	BBA69518	AF362160	AF362193	AF362254	AF362226
Fusarium oxysporum f. sp. *vasinfectum*	BBA69519	AF362157	AF362190	AF362251	AF362223
Fusarium oxysporum f. sp. *vasinfectum*	BBA69520	AF362140	AF362173	AF362234	AF362206
Fusarium oxysporum f. sp. *vasinfectum*	BBA69521	AF362139	AF362172	AF362233	AF362205
Fusarium oxysporum f. sp. *vasinfectum*	BBA69716	AF362163	AF362196	AF362257	AF362229
Fusarium oxysporum f. sp. *vasinfectum*	IMI-141112	EU246570	EU246604	EU246675	EU246641
Fusarium oxysporum f. sp. *vasinfectum*	AG86	EU246548	EU246595	EU246666	EU246632
Fusarium oxysporum f. sp. *vasinfectum*	ATCC 16611	EU246549	EU246596	EU246667	EU246633
Fusarium oxysporum f. sp. *vasinfectum*	BBA65635	AF362147	AF362180	AF362241	AF362213
Fusarium oxysporum f. sp. *vasinfectum*	BBA65636	AF362148	AF362181	AF362242	AF362214
Fusarium oxysporum f. sp. *vasinfectum*	BBA65653	AF362141	AF362174	AF362235	AF362207
Fusarium oxysporum f. sp. *vasinfectum*	BBA65655	AF362149	AF362182	AF362243	AF362215
Fusarium oxysporum f. sp. *vasinfectum*	BBA66844	AF362150	AF362183	AF362244	AF362216
Fusarium oxysporum f. sp. *vasinfectum*	BBA69405	AF362151	AF362184	AF362245	AF362217
Fusarium oxysporum f. sp. *vasinfectum*	SC1	EU246574	EU246608	EU246679	EU246645
Fusarium oxysporum f. sp. *vasinfectum*	BBA64495	AF362144	AF362177	AF362238	AF362210
Fusarium oxysporum f. sp. *vasinfectum*	BBA65633	AF362146	AF362179	AF362240	AF362212
Fusarium oxysporum f. sp. *vasinfectum*	BBA65634	AF362145	AF362178	AF362239	AF362211
Fusarium oxysporum f. sp. *vasinfectum*	BBA66847	AF362158	AF362191	AF362252	AF362224
Fusarium oxysporum f. sp. *vasinfectum*	BBA69711	AF362161	AF362194	AF362255	AF362227
Fusarium oxysporum f. sp. *vasinfectum*	IMI-141148	EU246571	EU246605	EU246676	EU246642
Fusarium oxysporum	3545	EU246540	EU246587	EU246657	EU246623
Fusarium oxysporum	3546	EU246541	EU246588	EU246658	EU246624
Fusarium oxysporum	3547	EU246542	EU246589	EU246659	EU246625
Fusarium oxysporum	3556	EU246543	EU246590	EU246661	EU246627
Fusarium oxysporum	6510	EU246545	EU246592	EU246663	EU246629
Fusarium oxysporum f. sp. *vasinfectum*	24492	EU246584	EU246618	EU246689	EU246655
Fusarium oxysporum f. sp. *vasinfectum*	24597	EU246580	EU246614	EU246685	EU246651
Fusarium oxysporum f. sp. *vasinfectum*	24598	EU246581	EU246615	EU246686	EU246652
Fusarium oxysporum	24646	EU246582	EU246616	EU246687	EU246653
Fusarium oxysporum f. sp. *vasinfectum*	B/96/02	EU246583	EU246617	EU246688	EU246654
Fusarium oxysporum	3608	EU246544	EU246591	EU246662	EU246628
Fusarium oxysporum f. sp. *vasinfectum*	24500	EU246575	EU246609	EU246680	EU246646
Fusarium oxysporum f. sp. *vasinfectum*	24595	EU246576	EU246610	EU246681	EU246647
Fusarium oxysporum f. sp. *vasinfectum*	41101	EU246578	EU246611	EU246682	EU246648
Fusarium oxysporum f. sp. *vasinfectum*	51101	EU246579	EU246612	EU246683	EU246649
Fusarium oxysporum	BW-2613	DQ435351	DQ435369	EU246656	EU246622
Fusarium oxysporum f. sp. *vasinfectum*	X1	EU246577	EU246613	EU246684	EU246650
Fusarium oxysporum	6632	EU246546	EU246593	EU246664	EU246630
Fusarium oxysporum	BW-3549	DQ435352	DQ435370	EU246660	EU246626
Fusarium oxysporum	NRRL25052	AF362171	AF362204	AF362260	AF362232

Table S4. *SIX6* gene sequences used for phylogenetic analysis in this study. They include these retrieved from a previous study (Rocha et al., 2016) as well as the ones generated in this study.

Species name	Isolate name	SIX6
Fusarium oxysporum f. sp. *vasinfectum*	SG1	PQ346567
Fusarium oxysporum f. sp. *vasinfectum*	SG55	PQ346568

Fusarium oxysporum f. sp. *vasinfectum*	TH1	PQ346569
Fusarium oxysporum f. sp. *vasinfectum*	SG26	PQ346570
Fusarium oxysporum f. sp. *vasinfectum*	RBG6378	KR855791
Fusarium oxysporum f. sp. *vasinfectum*	RBG5188	KR855790
Fusarium oxysporum f. sp. *vasinfectum*	RBG5186	KR855789
Fusarium oxysporum f. sp. *vasinfectum*	RBG5184	KR855788
Fusarium oxysporum f. sp. *vasinfectum*	FRL11157	KR855787
Fusarium oxysporum f. sp. *sesami*	FS08027	MN417209
Fusarium oxysporum f. sp. *radicis-cucumerinum*	RBG4743	KR855755
Fusarium oxysporum f. sp. *radicis-cucumerinum*	RBG4755	KR855754
Fusarium oxysporum f. sp. *passiflorae*	RBG6380	KR855782
Fusarium oxysporum f. sp. *passiflorae*	RBG5775	KR855781
Fusarium oxysporum f. sp. *pisi*	RBG6438	KR855778
Fusarium oxysporum f. sp. *pisi*	RBG6498	KR855777
Fusarium oxysporum f. sp. *niveum*	FRL1583	KR855756
Fusarium oxysporum f. sp. *niveum*	RBG5771	KR855744
Fusarium oxysporum f. sp. *lycopersici*	RBG5768	KR855786
Fusarium oxysporum f. sp. *cucumerinum*	RBG5106	KR855770
Fusarium oxysporum f. sp. *cucumerinum*	RBG5104	KR855769
Fusarium oxysporum	F114	MF314835
Fusarium oxysporum	F04	MF314833

Table S5. Spore concentrations of the liquid cultures determined on the day of inoculation.

Isolate name	Spore conc. (conidia/mL)[1]	Volume (mL)[1]	Spore conc. (conidia/mL)[2]	Volume (mL)[2]	Root dipping (conidia/mL)[3]	Volume (mL)[3]
Fov SG1	2.075×10^{7}	500	-	-	2.075×10^{7}	500
Fov SG26	2.7×10^{5}	500	-	-	2.7×10^{5}	500
Fov SG55	3.775×10^{7}	500	-	-	3.775×10^{7}	500
Fov TH1	2.45×10^{7}	500	-	-	2.45×10^{7}	500
RVB4.1	1.245×10^{6}	200	3.55×10^{6}	45	8.175×10^{5}	500
StrB22	1.25×10^{4}	200	2.5×10^{3}	45	5.225×10^{3}	500
BRR4	4.25×10^{4}	200	1.7825×10^{6}	45	1.77425×10^{5}	500
22BRR77	2.45×10^{6}	200	4.15×10^{6}	45	1.3535×10^{6}	500
Fo BRF1[4]	2.35×10^{7}	500	-	-	2.35×10^{7}	500
Fo BRF2[4]	2.0×10^{7}	500	-	-	2.0×10^{7}	500
Fo SHF6[4]	2.675×10^{7}	500	-	-	2.675×10^{7}	500
Fo WRF2[4]	6.7×10^{6}	500	-	-	6.7×10^{6}	500
Fov SG1[4]	3.375×10^{7}	500			3.375×10^{7}	500

[1] Liquid culture established using half-strength PDB (Fov and Fo) and 10% carrot extract (v/v) in sterile water (*B. rouxiae*). [2] Plate cultures using half-strength PDA (Fov and Fo) and 10% carrot extract (v/v) supplemented with 1.5% agar in sterile water (*B. rouxiae*). Five plates per *B. rouxiae* isolate were flooded with 10mL of sterile water. Spores and mycelia were collected and pooled totaling 45mL per isolate. [3] Spore concentrations of liquid and plate cultures combined in a total volume of 500mL. These cultures were used for root dipping. [4] This experiment was conducted at a later date

independently of the first experiment. Both experiments used the same growth chamber and the same set of growth parameters.

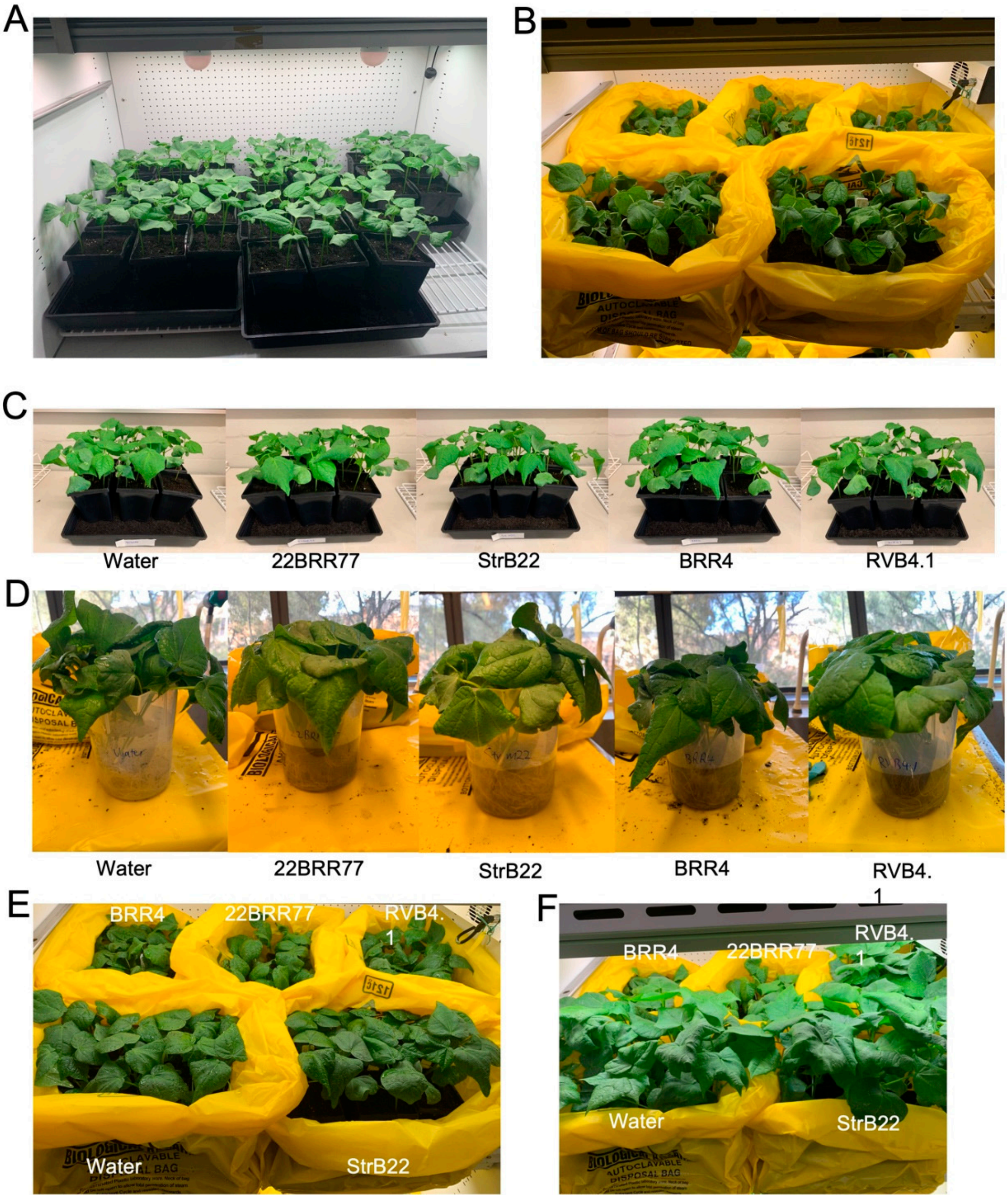

Figure S1. Plant growth before and after inoculation with *Berkeleyomyces rouxiae* isolates. **(A)** 12 days old Sicot746 B3F seedlings sitting in a double-tier growth chamber. **(B)** 14 days old Sicot746 B3F seedlings immediately after inoculation. **(C)** 14 days old Sicot746 B3F seedlings before inoculation. **(D)** Root dipping was performed using unfiltered cultures. **(E)** Sicot746 B3F seedlings were recovering in the growth chamber at 4 days post inoculation (dpi). **(F)** Sicot746 B3F seedlings were recovering in the growth chamber at 12 dpi.

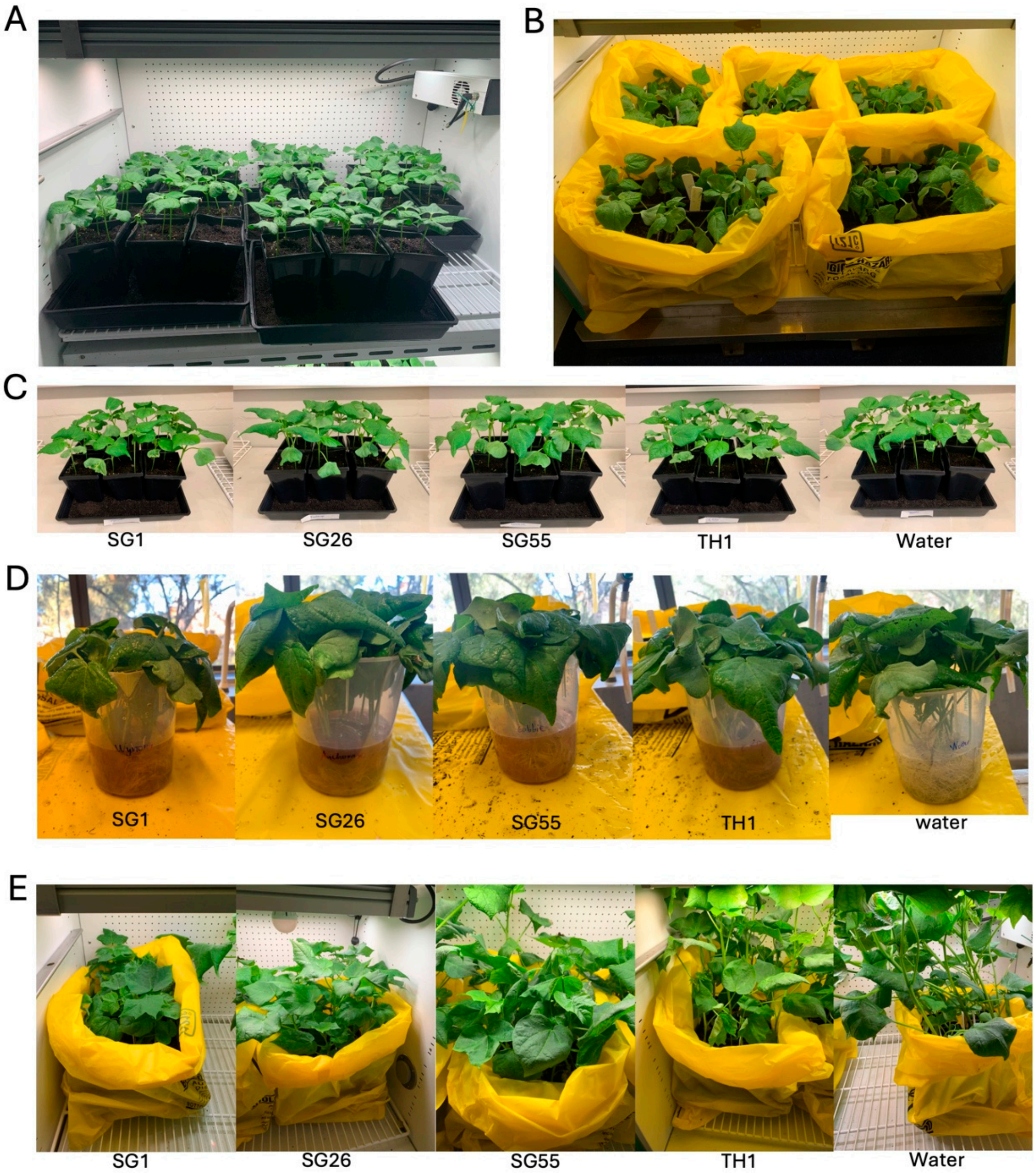

Figure S2. Plant growth before and after inoculation with *Fusarium oxysporum* f. sp. *vasinfectum* isolates. **(A)** 12 days old Sicot746 B3F seedlings sitting in a double-tier growth chamber. **(B)** 14 days old Sicot746 B3F seedlings immediately after inoculation. **(C)** 14 days old Sicot746 B3F seedlings before inoculation. **(D)** Root dipping was performed using unfiltered cultures. **(E)** Sicot746 B3F seedlings were recovering in the growth chamber at 19 days post inoculation.

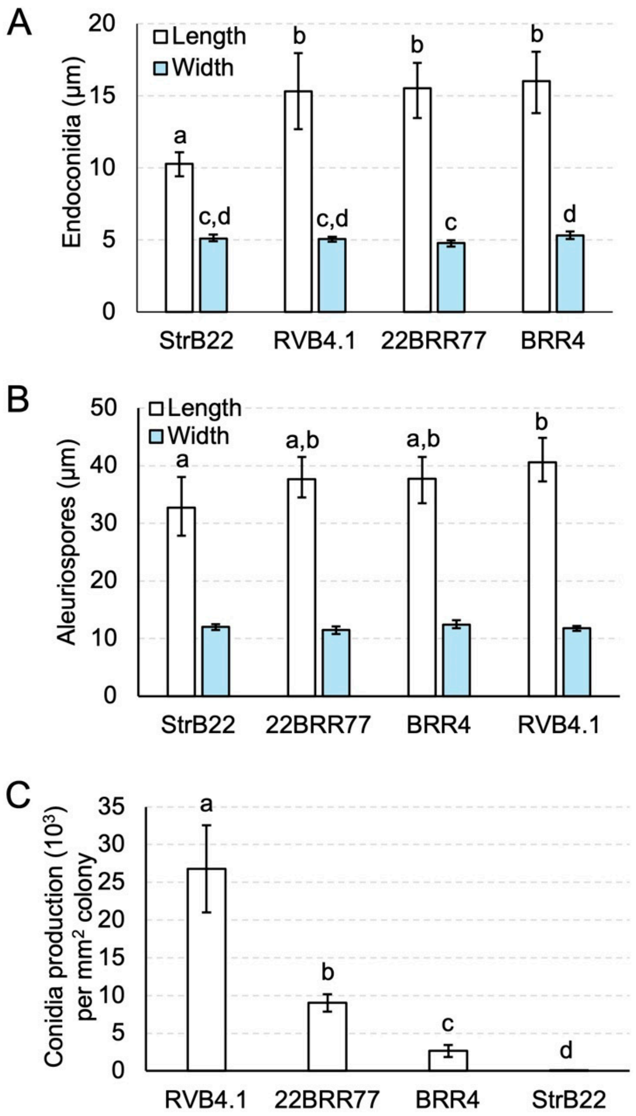

Figure S3. The spore size and growth rates of *Berkeleyomyces rouxiae* isolates after 3 weeks of growth on half-strength PDA plates. **(A)** Endoconidia size (n = 12). **(B)** Aleuriospore size (n = 12). **(C)** Growth rates in conidia production per mm^2 colony (n = 6). Letters indicate separation of means by Tukey's range test at p = 0.05. Error bars indicate a 95% confidence interval.

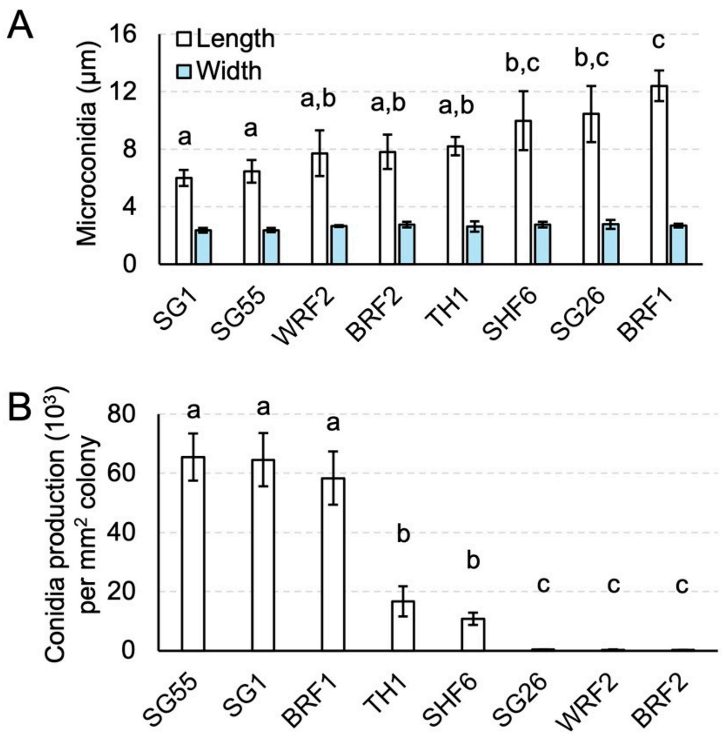

Figure S4. The spore size and growth rates of *Fusarium oxysporum* isolates after seven days of growth on half-strength PDA plates. **(A)** Microconidia size (n = 12). **(B)** Growth rates in conidia production per mm^2 colony (n = 5). Letters indicate significant separation of means by Tukey's range test at p = 0.05. Error bars indicate a 95% confidence interval.

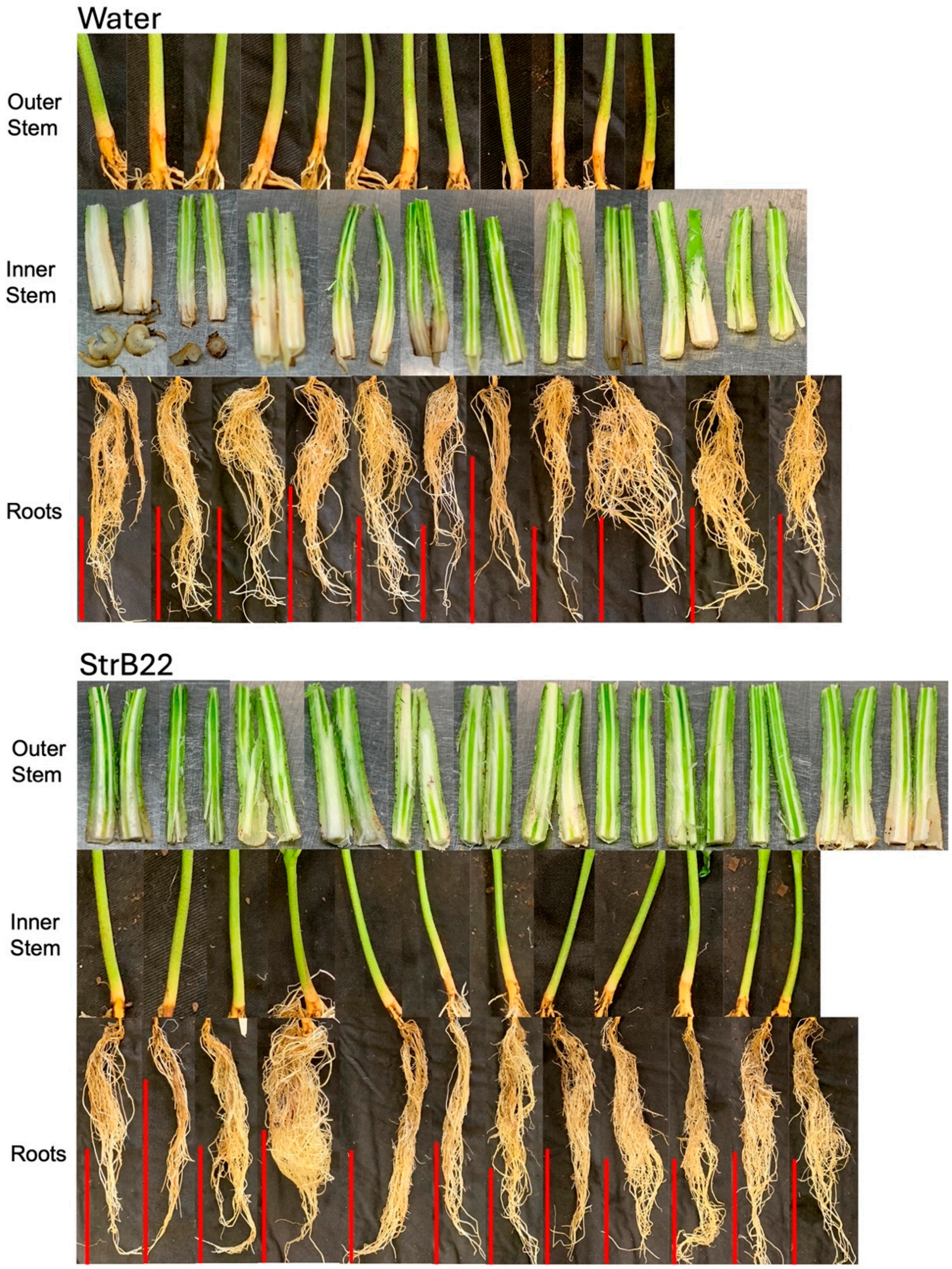

Figure S5. Symptomatology of Sicot746 B3F seedlings inoculated with *Berkeleyomyces rouxiae* at harvest (15 — 20 days post inoculation). Uninoculated controls and plants inoculated with StrB22 are shown. Red vertical bars indicate a scale of 10 cm.

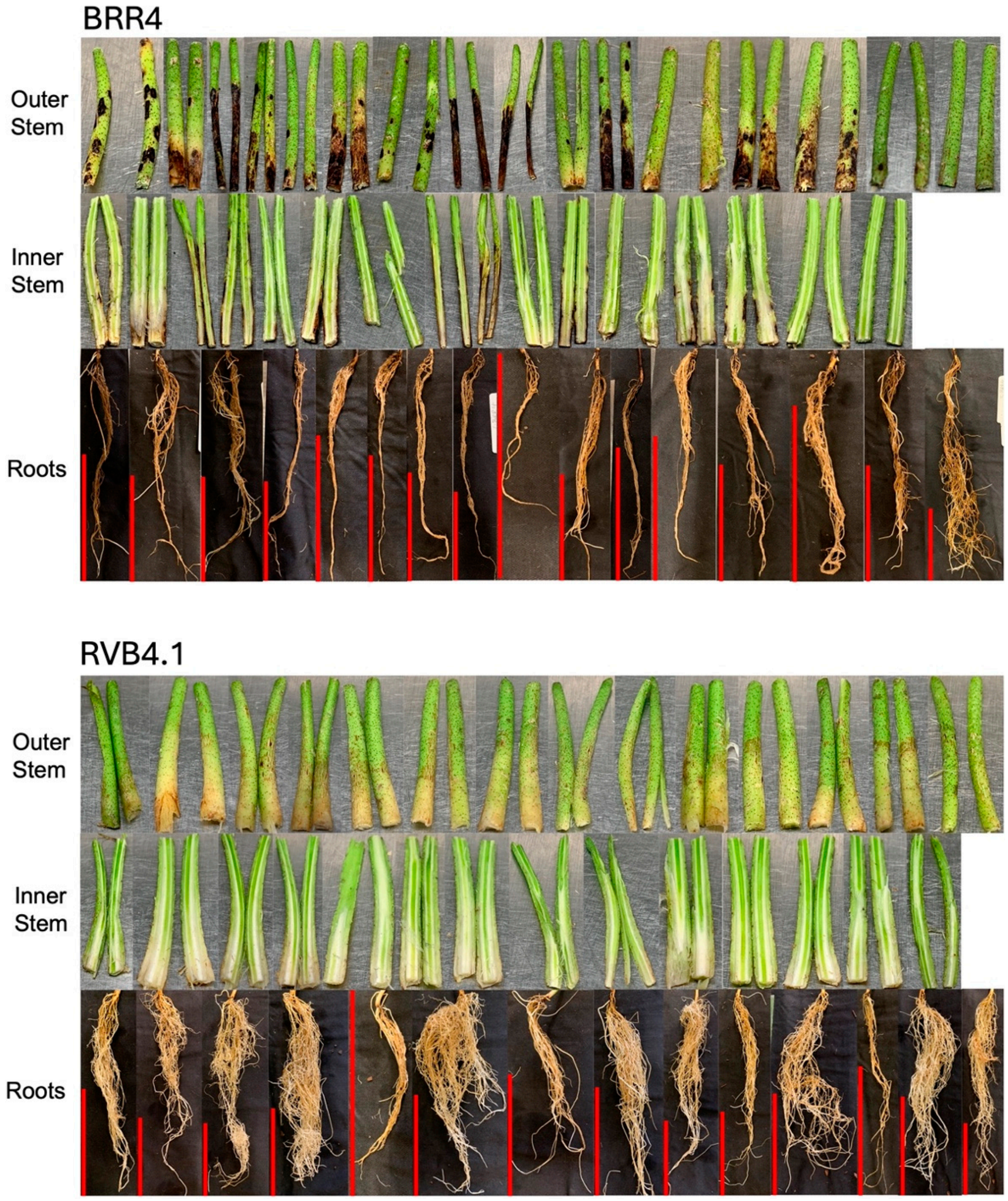

Figure S6. Symptomatology of Sicot746 B3F seedlings inoculated with *Berkeleyomyces rouxiae* at harvest (15 — 20 days post inoculation). Plants inoculated with BRR4 and RVB4.1 are shown. Red vertical bars indicate a scale of 10 cm.

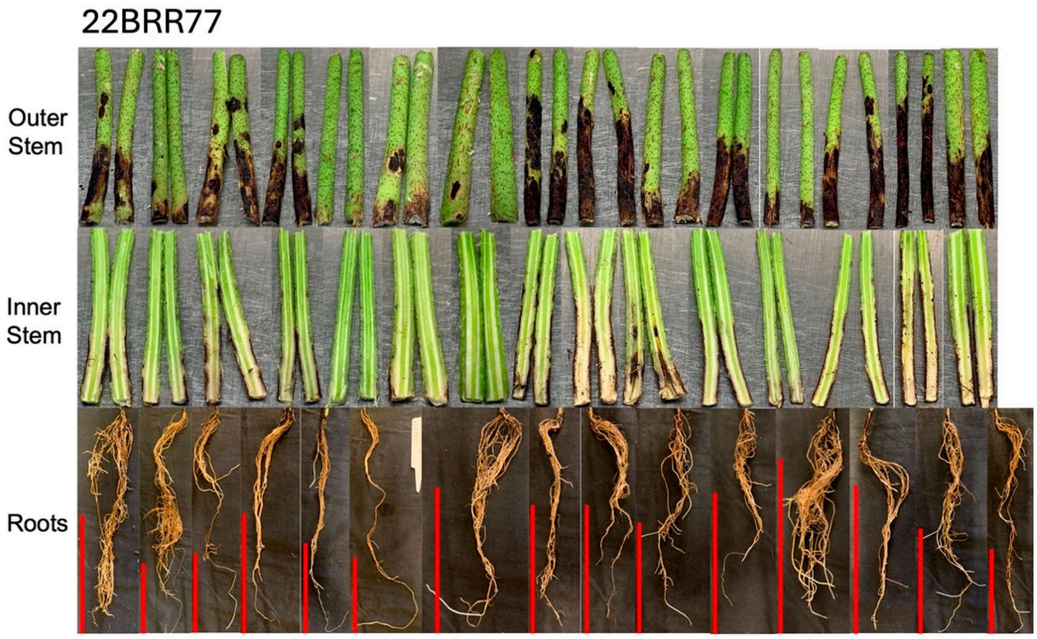

Figure S7. Symptomatology of Sicot746 B3F seedlings inoculated with *Berkeleyomyces rouxiae* at harvest (15 — 20 days post inoculation). Plants inoculated with 22BRR77 are shown. Red vertical bars indicate a scale of 10 cm.

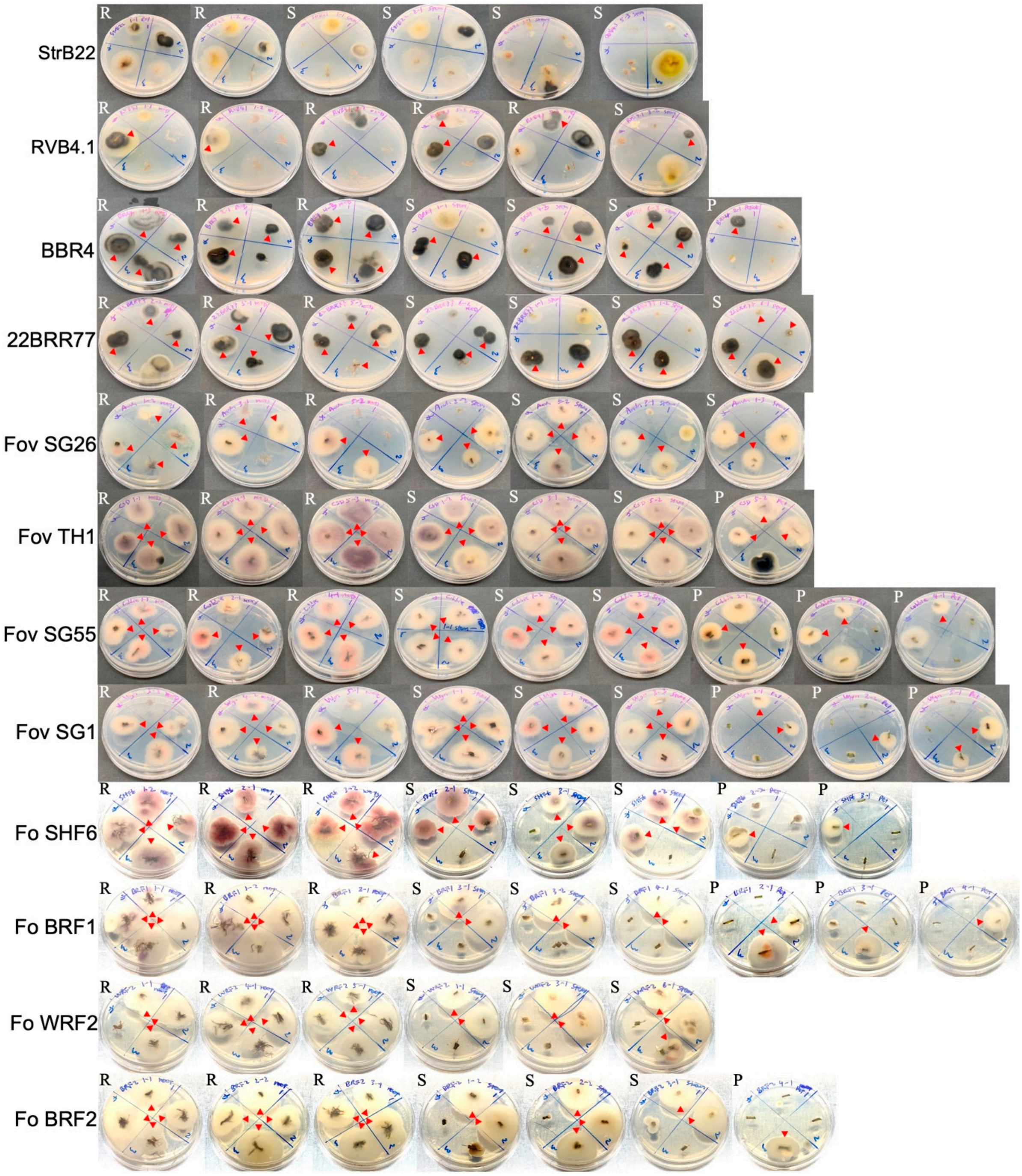

Figure S8. Reisolation of the *Berkeleyomyces rouxiae* and *Fusarium oxysporum* isolates from cotton plants on half-strength PDA plates (reverse view). Red arrows indicate *B. rouxiae* and *F. oxysporum* morphology positively identified under a compound microscope. R = roots; S = stem; P = petiole.

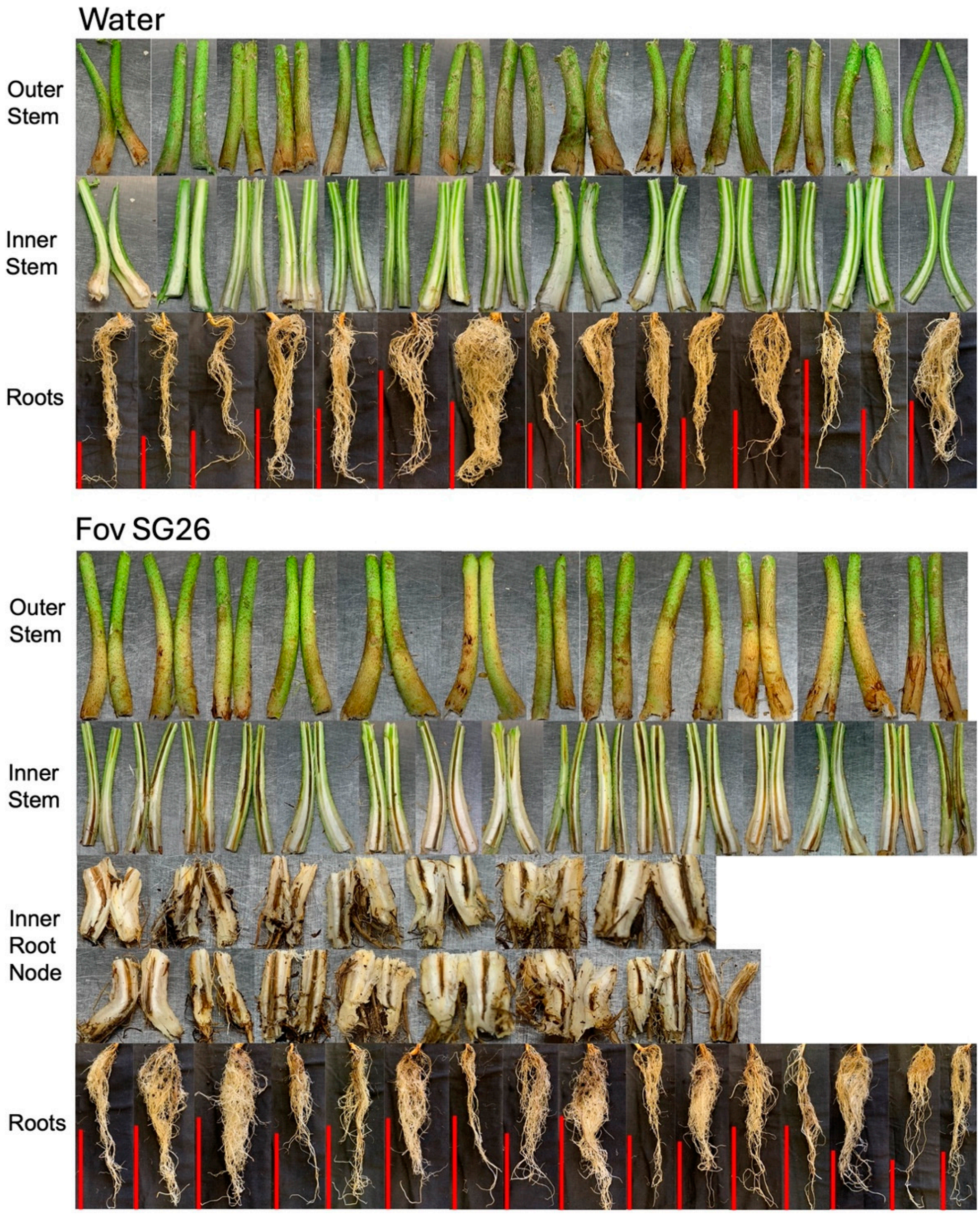

Figure S9. Symptomatology of Sicot746 B3F seedlings inoculated with *Fusarium oxysporum* f. sp. *vasinfectum* at harvest (27 — 34 days post inoculation). Uninoculated controls and Plants inoculated with Fov SG26 are shown. Red vertical bars indicate a scale of 10 cm.

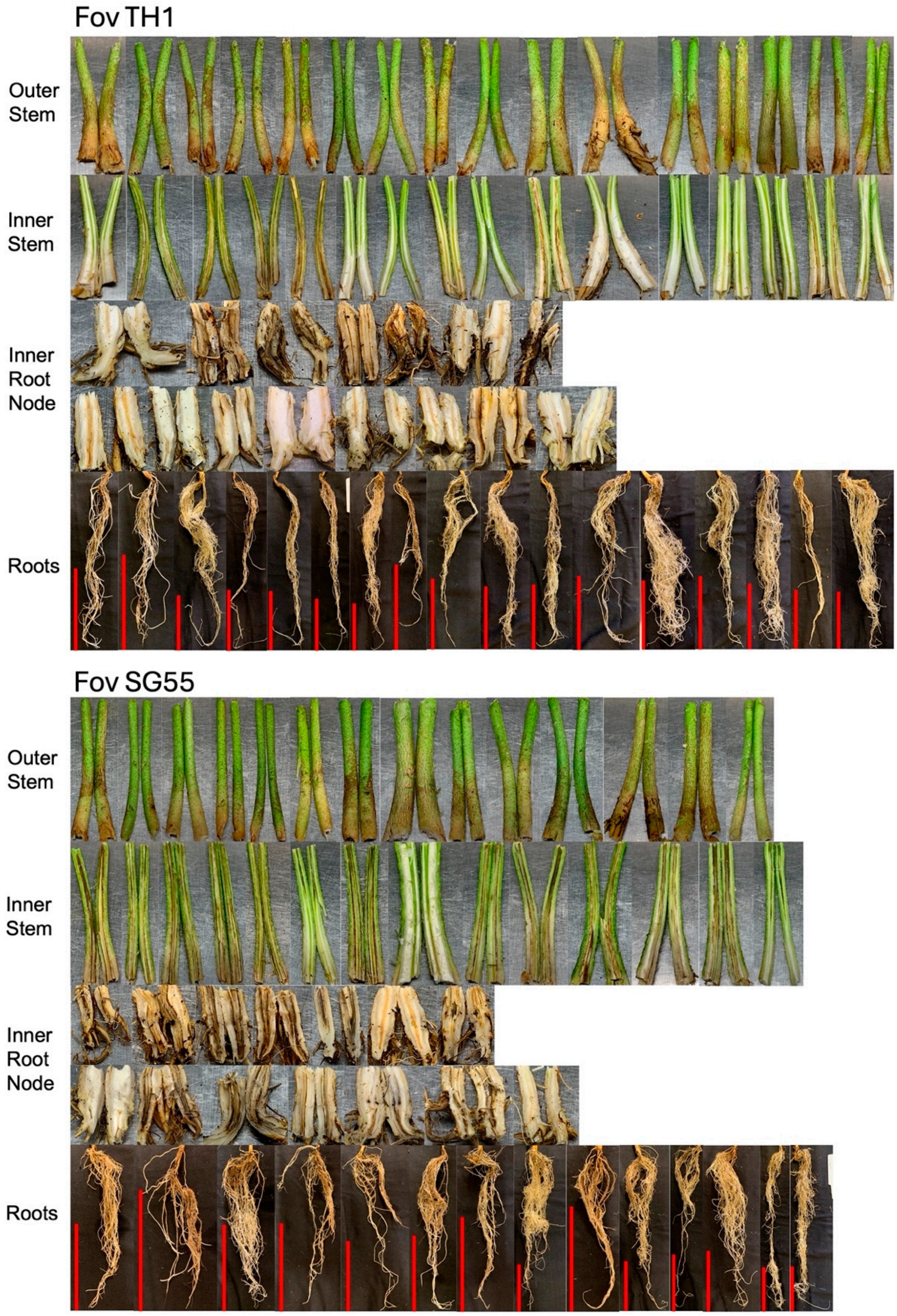

Figure S10. Symptomatology of Sicot746 B3F seedlings inoculated with *Fusarium oxysporum* f. sp. *vasinfectum* at harvest (27 — 34 days post inoculation). Plants inoculated with Fov TH1 and Fov SG55 are shown. Red vertical bars indicate a scale of 10 cm.

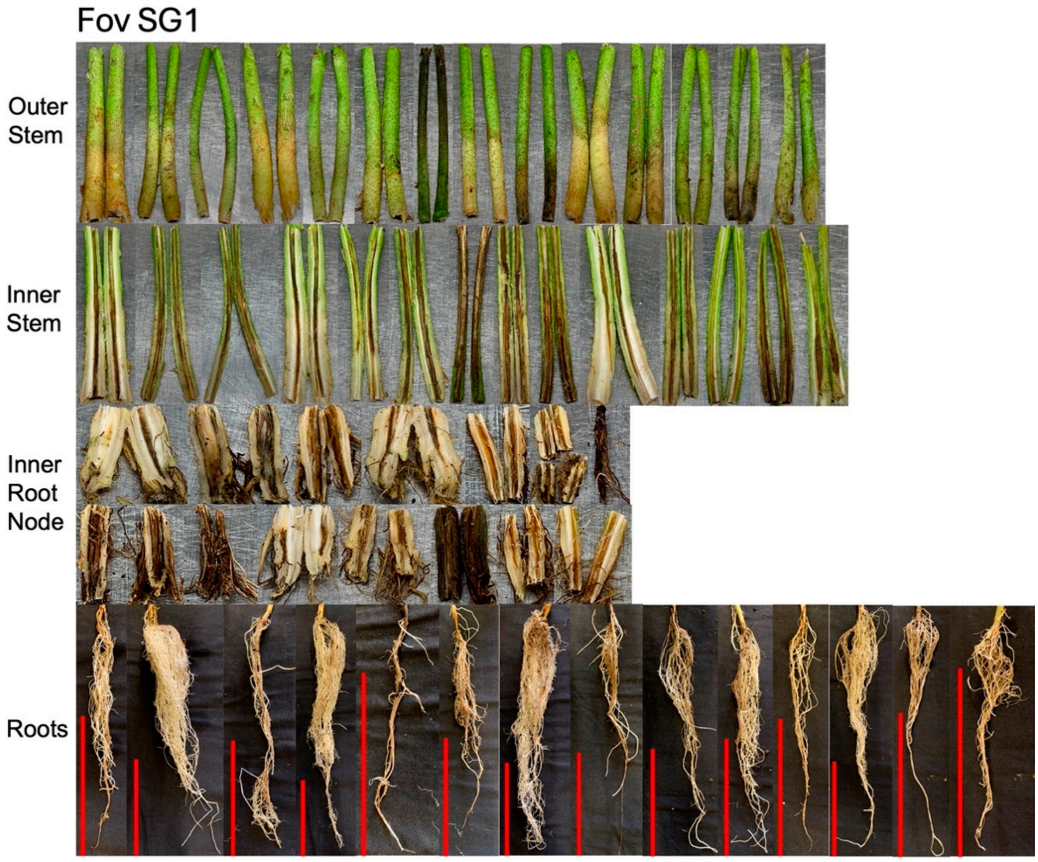

Figure S11. Symptomatology of Sicot746 B3F seedlings inoculated with *Fusarium oxysporum* f. sp. *vasinfectum* at harvest (27 — 34 days post inoculation). Plants inoculated with Fov SG1 are shown. Red vertical bars indicate a scale of 10 cm.

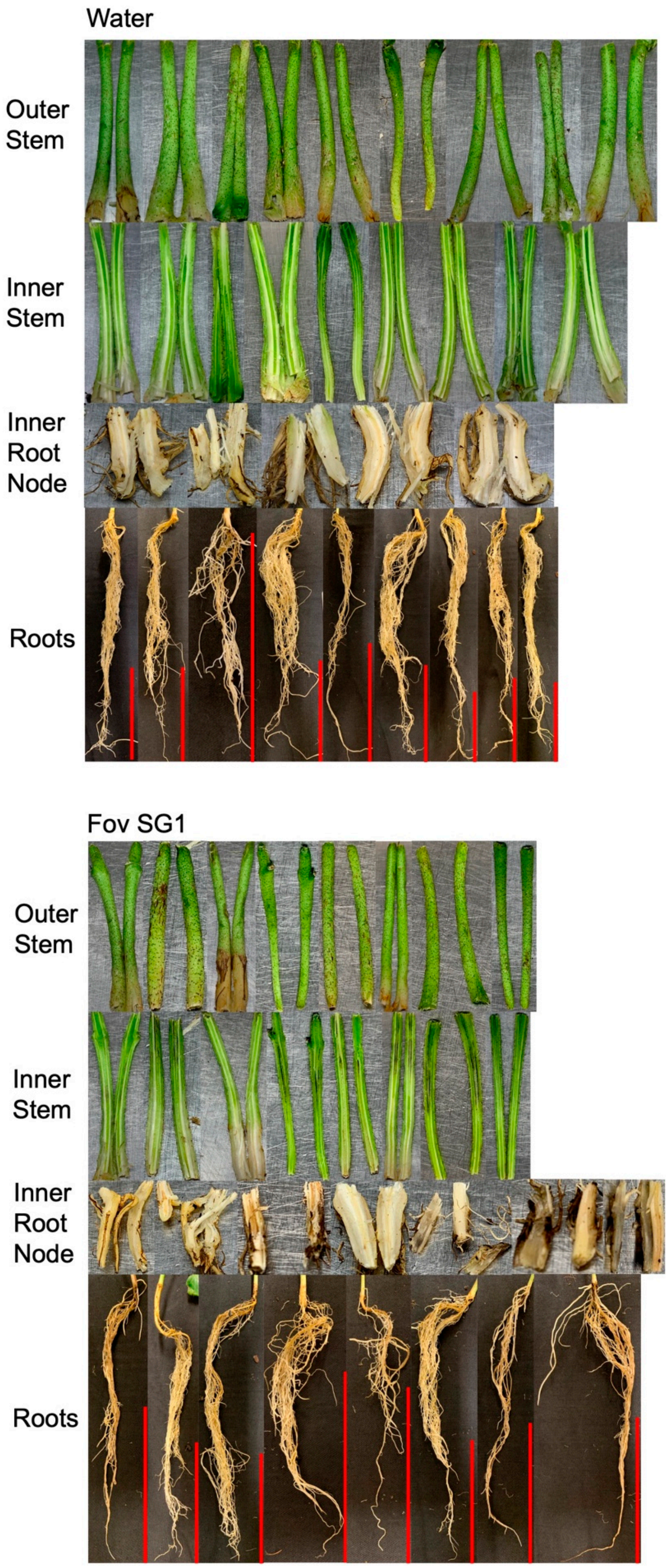

Figure S12. Symptomatology of Sicot746 B3F seedlings inoculated with *Fusarium oxysporum* spp. at harvest (17 — 22 days post inoculation). Uninoculated plants and plants inoculated with Fov SG1 (positive controls) are shown. Red vertical bars indicate a scale of 10 cm.

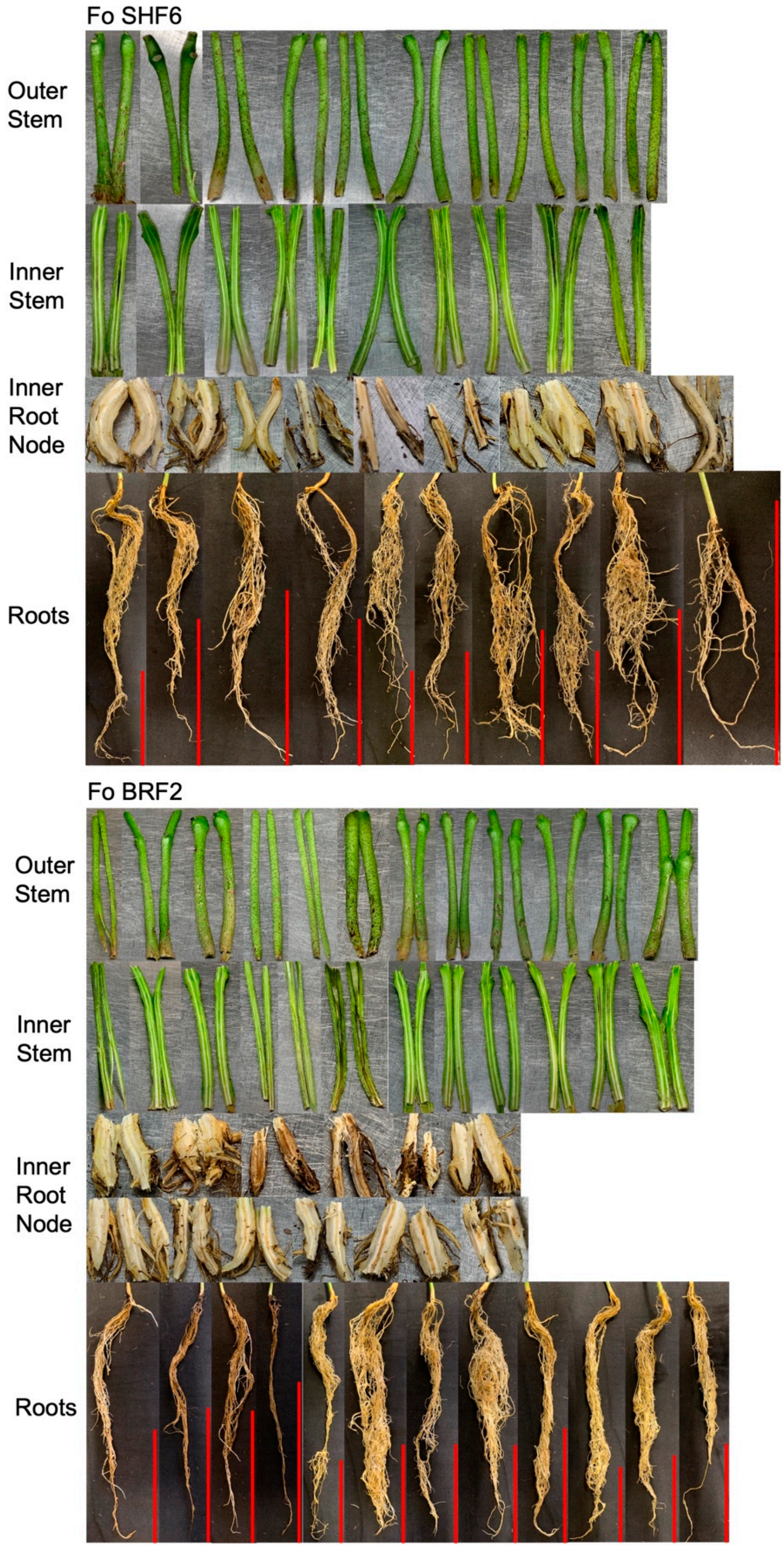

Figure S13. Symptomatology of Sicot746 B3F seedlings inoculated with *Fusarium oxysporum* spp. at harvest (17 — 22 days post inoculation). Plants inoculated with Fo SHF6 and Fo BRF2 are shown. Red vertical bars indicate a scale of 10 cm.

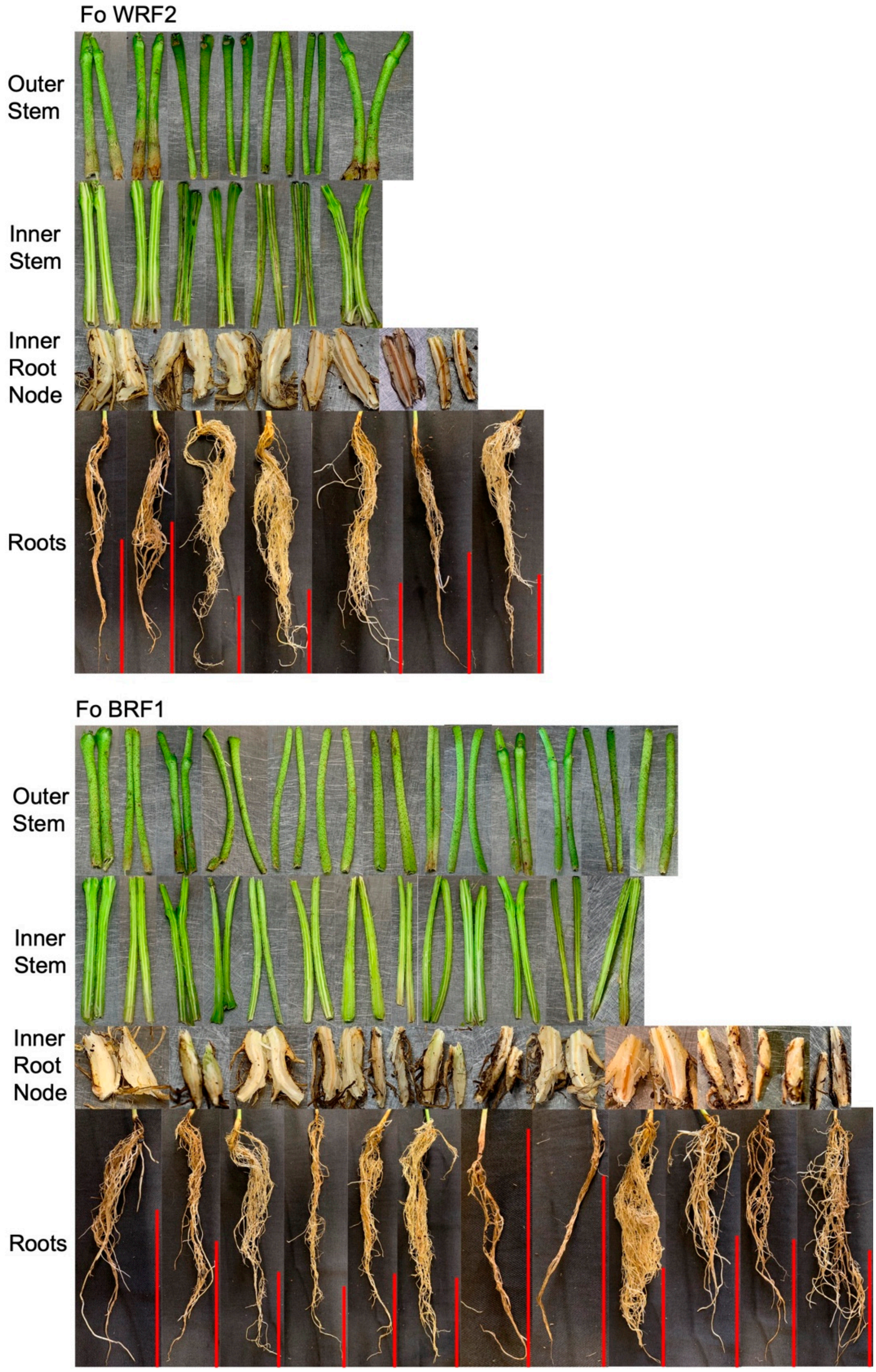

Figure S14. Symptomatology of Sicot746 B3F seedlings inoculated with *Fusarium oxysporum* spp. at harvest (17 — 22 days post inoculation). Plants inoculated with Fo WRF2 and Fo BRF1 are shown. Red vertical bars indicate a scale of 10 cm.
